# Prescribing Trajectories in Type 2 Diabetes in the United States, 2019–2024

**DOI:** 10.1111/dom.71013

**Published:** 2026-06-17

**Authors:** Tobias S. Lux, Dongkun Lee, Jeff M. Phillips, Julio C. Facelli, Daniel C. Malone, Matthew Wahl, Deepika Reddy, Farrant Sakaguchi, Ramkiran Gouripeddi, Alexander S. Millar, Matthew J. O'Brien, Joshua S. Choi, Jittrapol Sutejitsiri, Kensaku Kawamoto, Polina V. Kukhareva

**Affiliations:** ^1^ Kahlert School of Computing, University of Utah Salt Lake City Utah USA; ^2^ Department of Biomedical Informatics University of Utah Salt Lake City Utah USA; ^3^ Utah Clinical and Translational Institute, University of Utah Salt Lake City Utah USA; ^4^ Department of Pharmacotherapy University of Utah Salt Lake City Utah USA; ^5^ Diabetes and Endocrinology Center University of Utah Salt Lake City Utah USA; ^6^ Department of Family and Public Health University of Utah Salt Lake City Utah USA; ^7^ Department of General Internal Medicine Northwestern University Chicago Illinois USA

**Keywords:** cohort study, database research, GLP‐1, incretin therapy, pharmaco‐epidemiology, population study

## Abstract

**Importance:**

Clinical guidelines for type 2 diabetes (T2D) provide population‐level recommendations, but real‐world treatment patterns evolve dynamically and vary across patients. Understanding longitudinal prescribing trajectories may reveal heterogeneity in care not captured by cross‐sectional analyses.

**Objective:**

To identify and characterise real‐world T2D prescribing trajectories using nationwide electronic health record (EHR) data.

**Design, Setting and Participants:**

This retrospective cohort study used de‐identified EHR data from the TriNetX Research Network. Adults newly diagnosed with T2D who initiated their first glucose‐lowering medication in the first half of 2019 were followed from 2019 through 2024.

**Exposures:**

Longitudinal glucose‐lowering prescribing trajectories derived from prescription orders across nine drug classes (metformin, sulfonylureas, thiazolidinediones, dipeptidyl peptidase‐4 inhibitors, sodium‐glucose cotransporter 2 inhibitors, glucagon‐like peptide‐1 RAs (GLP‐1 RAs), dual glucose‐dependent insulinotropic polypeptide/GLP‐1 RAs (GIP/GLP‐1 RAs), insulin and other antidiabetic agents), encoded in 12 semiannual intervals and identified using hierarchical agglomerative clustering.

**Main Outcomes and Measures:**

Prescribing trajectory cluster membership; adjusted longitudinal changes in body mass index (BMI) and haemoglobin A1c (HbA1c); and associations between cluster membership and patient characteristics assessed using multivariable logistic regression.

**Results:**

Among 9327 patients, 30 distinct prescribing trajectory clusters were identified and grouped into monotherapy, dual therapy, complex therapy, variant therapy and GLP‐1 RA‐based trajectories. Treatment intensification patterns, BMI and HbA1c trajectories and demographic composition varied substantially across clusters. Within GLP‐1 RA–based trajectories (11 clusters), substantial within‐group reductions in BMI and HbA1c were observed; these clusters generally comprised patients with higher baseline BMI and most commonly involved transitions following metformin. GLP‐1 RA prescribing was more frequent among younger patients and those with higher baseline BMI. In early GLP‐1 RA transition trajectories, Asian and Hispanic patients had lower odds of cluster membership compared with non‐Hispanic White patients (Asian: odds ratio [OR] 0.40; 95% CI 0.23–0.63; Hispanic: OR 0.57; 95% CI 0.40–0.79).

**Conclusions and Relevance:**

Data‐driven clustering of longitudinal EHR medication data identifies substantial heterogeneity in real‐world T2D treatment trajectories. Differences in the timing and adoption of newer therapies were observed across demographic groups, underscoring the value of longitudinal approaches for evaluating real‐world diabetes care patterns.

## 1 Introduction

Diabetes affects more than 38 million people in the United States and is a leading cause of cardiovascular disease, kidney failure and neuropathy [1]. Clinical practice guidelines provide evidence‐based recommendations for diabetes management but are largely derived from randomised clinical trial populations and may not fully reflect real‐world variation in patient characteristics, comorbidities and care settings [2, 3]. As the prevalence of type 2 diabetes (T2D) continues to increase and newer glucose‐lowering therapies, including glucagon‐like peptide‐1 receptor agonists (GLP‐1 RAs), have become available, understanding how these treatments are used in routine clinical practice has become increasingly important [4, 5].

The real‐world adoption of newer T2D therapies is influenced by multiple factors, including insurance coverage, out‐of‐pocket costs and prescribing practices [6]. Prior studies have demonstrated variation in diabetes treatment patterns by demographic and clinical characteristics. Younger adults are more likely to receive early combination therapy, men are more likely to receive newer agents and Black patients are less likely than White patients to receive GLP‐1 RAs, sodium‐glucose cotransporter 2 (SGLT2) inhibitors, or timely treatment intensification [7, 8].

National electronic health record (EHR) databases enable analysis of real‐world treatment patterns at scale and frequently reveal differences from trial‐based evidence [9, 10]. For example, EHR‐based studies have reported higher rates of GLP‐1 RA discontinuation than observed in clinical trials, highlighting gaps between guideline recommendations and real‐world use [11]. These findings underscore the importance of examining longitudinal treatment patterns as they occur in routine care.

Although prior studies using EHR or claims data have characterised trends in medication use for T2D, most have focused on individual drug classes or cross‐sectional uptake rather than longitudinal treatment trajectories [12, 13]. Such approaches may obscure the dynamic nature of diabetes management, including dynamic treatment regimens based on HbA1c level changes, treatment escalation, switching, combination therapy and discontinuation. Preserving the temporal sequence of medication use may provide a more comprehensive understanding of real‐world prescribing behaviour [14]. The objective of this study was to characterise real‐world T2D prescribing trajectories using data‐driven clustering methods applied to a large, national EHR database.

## 2 Methods

### 2.1 Study Design

We conducted a retrospective cohort study using de‐identified EHR data to characterise longitudinal prescribing trajectories among adults newly diagnosed with T2D. The study followed the Reporting of Studies Conducted Using Observational Routinely Collected Health Data (RECORD) guidelines [15]. The University of Utah IRB determined that this study constituted non‐human subjects research (#00175012).

### 2.2 Data Source

TriNetX is a federated research network containing de‐identified EHR data from more than 100 health care organisations, representing approximately 130 million patients in the United States [16, 17]. The database includes longitudinal diagnoses, procedures, medications and laboratory results. The dataset used in this study was provided to the University of Utah by the TriNetX Research Network in May 2025.

### 2.3 Study Population

Cohort construction is shown in Figure S1 and inclusion and exclusion codes are provided in Table S1. Diagnoses were identified using ICD‐10‐CM codes, medications using ATC and RxNorm codes and laboratory results using LOINC terminology.

We included adults (≥ 18 years) whose first recorded T2D diagnosis (ICD‐10‐CM E11.*) and first glucose‐lowering medication prescription both occurred between January and June 2019. Requiring concordant diagnosis and medication evidence is consistent with a validated EHR‐based computable phenotype for T2D identification with a positive predictive value of 96.4% [18]. To reduce misclassification of prevalent disease as incident disease, patients were required to have at least one encounter before January 1, 2018. We excluded patients with any T2D diagnosis before July 1, 2018, any glucose‐lowering medication prescription before 2019, or evidence of pre‐existing hyperglycemia defined as ≥ 2 HbA1c values > 6.5% before July 1, 2018, consistent with the SUPREME‐DM phenotype [19]. Additional exclusions included type 1 diabetes, gestational or postpartum diabetes, missing or unknown sex and residence outside the United States.

We also excluded 10 clusters (*N* = 9325) identified by the clustering algorithm with sparse longitudinal prescribing activity, consistent with early disengagement from care or transition outside the TriNetX network. Because trajectory analyses require sustained follow‐up, these clusters were analysed separately and excluded from downstream outcome analyses (Figure S2). The remaining 30 clusters formed the primary analytic cohort. The main difference between the primary analytic cohort and the disengagement cohort was by age over 65, potentially showing that patients switch to Medicare and change their primary care provider (Table S2). Sensitivity analyses using patient‐level sparsity filtering produced similar therapy group classifications (Figure S3).

### 2.4 Patient Characteristics

Demographic variables included age in 2019 (< 55, 55–64, ≥ 65 years), sex, race and ethnicity and geographic region. Race and ethnicity were categorised as non‐Hispanic White, non‐Hispanic Black, non‐Hispanic Asian, Hispanic/Latinx and Other. Baseline clinical variables included BMI, HbA1c, heart failure (HF) and chronic kidney disease (CKD) measured in the beginning of the study (2019). BMI was categorised as < 25, 25–30, ≥ 30 kg/m^2^, or missing; HbA1c as < 7%, ≥ 7%, or missing. A composite HF/CKD indicator was created.

### 2.5 Medication Prescribing

Medication prescribing was defined using prescription orders for 9 glucose‐lowering medication classes: metformin, sulfonylureas, thiazolidinediones, DPP‐4 inhibitors, SGLT2 inhibitors, GLP‐1 RAs, dual GIP/GLP‐1 RAs, insulin and other antidiabetic agents (i.e., acarbose, miglitol, nateglinide, pramlintide, repaglinide). Initial therapy was categorised as monotherapy, dual therapy or complex therapy if patients were prescribed 1, 2 or ≥ 3 medication classes, respectively, during the first half of 2019.

### 2.6 Prescribing Trajectory Construction

Each patient's 6 year (2019–2024) medication history was divided into twelve 6‐month intervals from January 2019 through December 2024. For each interval, each medication class was coded as binary (1 if prescribed during the interval, 0 otherwise), yielding a 12 × 9 medication matrix per patient. This class‐level binary representation follows prior sequence‐based EHR trajectory clustering [20].

Six‐month intervals balance clinical granularity with analytic tractability: the American Diabetes Association recommends reassessing glycemic control and adjusting pharmacotherapy approximately every 3 to 6 months, aligning the semiannual interval with routine diabetes management cycles. Robustness of cluster assignments to this binning choice was confirmed in a sensitivity analysis using 3‐month bins (Figure S4).

Trajectories were aligned by calendar time rather than time since diagnosis to capture calendar trends in diabetes treatment, including changes in guidelines, drug availability, insurance coverage and rapid adoption of newer agents. Robustness of cluster assignments to this alignment choice was confirmed in a sensitivity analysis re‐anchored to each patient's first prescription date (Figure S5). Each patient's medication matrix was flattened into a single vector for clustering (Figure S6) [20].

### 2.7 Clustering Methodology

We applied unsupervised hierarchical agglomerative clustering to patient‐level medication vectors to identify common longitudinal prescribing patterns. Four linkage methods (single, average, complete and Ward) were compared; Ward's was further evaluated across *k* = 2 to 50. Ward linkage, which minimises within‐cluster variance using squared Euclidean distance, produced the most coherent and clinically interpretable clusters (Figure S7). Cluster quality was assessed using within‐cluster sum of squares, silhouette coefficients and the gap statistic (Figure S8) [21, 22, 23]. Final selection of *k* = 40 was guided by these diagnostics together with visual review of partitions at *k* = 20, 30, 40 and 50 (Figure S9), balancing partition stability with sufficient granularity to distinguish clinically meaningful prescribing patterns.

### 2.8 Cluster Categories

Derived clusters were visually inspected and feedback was obtained from practicing endocrinologists and primary care clinicians to ensure clinical interpretability. Clusters were grouped into higher‐level categories based on dominant prescribing patterns as GLP‐1 RA–based, monotherapy, dual therapy, complex therapy, or variant therapy. Each patient was assigned to a single cluster.

### 2.9 Statistical Analysis

Prescribing trajectories were visualised alongside adjusted BMI and HbA1c trends using 6‐month means with 95% confidence intervals. Adjusted BMI and HbA1c trajectories were estimated using linear mixed‐effects models with patient‐level random intercepts for the subset of patients with at least one recorded value in every calendar year from 2019 through 2024 (HbA1c‐eligible patients contributed a median of 13 measurements [IQR 11–15]; BMI‐eligible patients contributed a median of 30 measurements [IQR 20–46]); when multiple measurements were recorded within a single calendar year, the arithmetic mean served as that patient's annual value. Models included fixed effects for age (linear and quadratic terms), calendar year, cluster, sex, race and ethnicity, geographic region and cluster‐by‐year interactions. Adjusted means were estimated using marginal effects at the mean baseline age.

Associations between patient characteristics and cluster membership were evaluated using multivariable logistic regression in a one‐vs.‐rest framework. Covariates included race and ethnicity, age category, sex, baseline BMI category, baseline HbA1c category and baseline HF/CKD status. Results are reported as adjusted odds ratios with 95% confidence intervals. Because analyses were exploratory, we report both unadjusted *p*‐values and Benjamini–Hochberg (BH) false discovery rate‐adjusted *p*‐values. Analyses were conducted using PostgreSQL version 16.1, Python version 3.11.13 and R version 4.4.0. Code used in this study is publicly available at https://github.com/Kukhareva‐Lab/t2d‐prescribing‐trajectories.

## 3 Results

### 3.1 Cohort Characteristics and Cluster Overview

The final analytic cohort included 9327 patients across 30 prescribing trajectory clusters (Table 1), including five monotherapy, eight dual‐therapy, three complex‐therapy, three variant‐therapy and 11 GLP‐1 RA–based clusters. A practical summary of cluster‐level prescribing patterns, dominant drug classes and observed BMI/HbA1c trends is provided in Table S3. Baseline demographics and comorbidities are summarised in Table S4, with adjusted HF and CKD rates by cluster shown in Table S5. Patient counts used for adjusted BMI and HbA1c trajectories are shown in Table S6. Baseline differences across race/ethnicity in age, BMI, HbA1c and HF/CKD prevalence are presented in Table S7.

**TABLE 1:** Patient characteristics.

Patient characteristics	Summary
*N*	9327
Demographics	
Female sex	*n* = 4484, 48.1%
Age category, years	
< 55	*n* = 3894, 41.7%
55–64	*n* = 2923, 31.3%
≥ 65	*n* = 2510, 26.9%
Race/ethnicity	
African American/Black, Non‐Hispanic	*n* = 1657, 17.8%
Asian, Non‐Hispanic	*n* = 605, 6.5%
Hispanic/Latinx	*n* = 863, 9.3%
White, Non‐Hispanic	*n* = 5686, 61.0%
Other, Non‐Hispanic	*n* = 516, 5.5%
US Region	
Midwest	*n* = 2000, 21.4%
South	*n* = 3269, 35.0%
West	*n* = 1255, 13.5%
Northeast	*n* = 2803, 30.1%

Clinical characteristics	Summary
Baseline HbA1c (pre‐treatment, 2019, %)	
≤ 6.4	*n* = 502, 5.4%
6.5–7.9	*n* = 2701, 29.0%
8.0–8.9	*n* = 656, 7.0%
≥ 9.0	*n* = 1658, 17.8%
Missing	*n* = 3810, 40.8%
Baseline BMI (pre‐treatment, 2019, kg/m^2^)	
≤ 24.9	*n* = 262, 2.8%
25.0–29.9	*n* = 885, 9.5%
30.0–34.9	*n* = 1335, 14.3%
35.0–39.9	*n* = 1030, 11.0%
≥ 40	*n* = 990, 10.6%
Missing	*n* = 4825, 51.7%
Comorbidities before June 1, 2019	
Heart failure	*n* = 574, 6.2%
Chronic kidney disease	*n* = 710, 7.6%

*Note:* Values are summarised as *N* (%). Baseline glycated haemoglobin (HbA1c) and body mass index (BMI) represent the closest recorded value at or before each patient's first glucose‐lowering medication (GLM) prescription, restricted to a 183‐day pre‐treatment window within 2019. Patients without a qualifying pre‐treatment value are reported as missing. Comorbidities indicate documented heart failure (HF) or chronic kidney disease (CKD) between January 1 and June 1, 2019.

**FIGURE 1:**
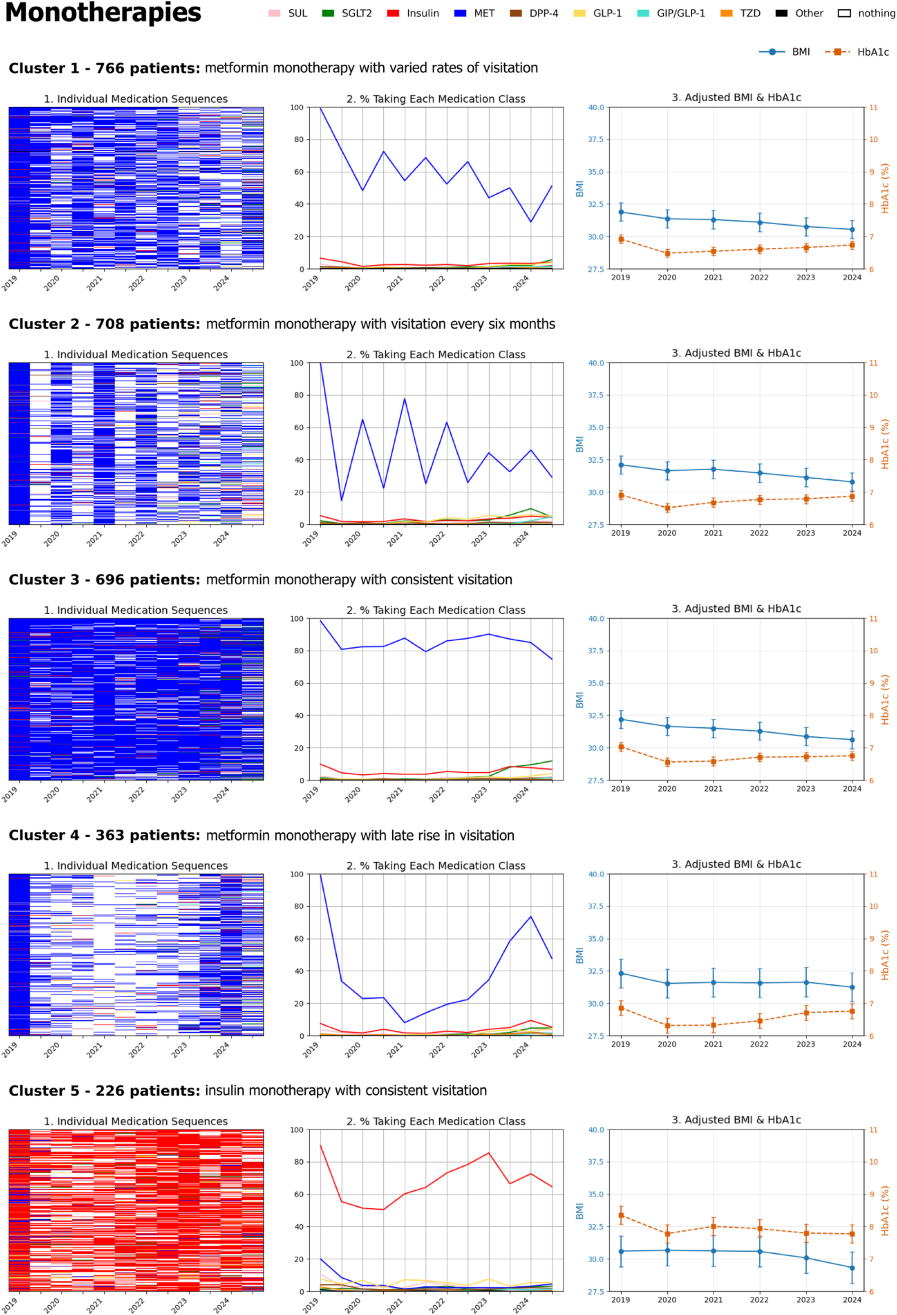
Monotherapy clusters visualisations. This figure presents three‐panel visualisations for all clusters classified within the Monotherapy group. Each panel includes (1) individual patient trajectory heatmaps, (2) medication prescribing distributions over time and (3) adjusted BMI and HbA1c trajectories where available.

**FIGURE 2:**
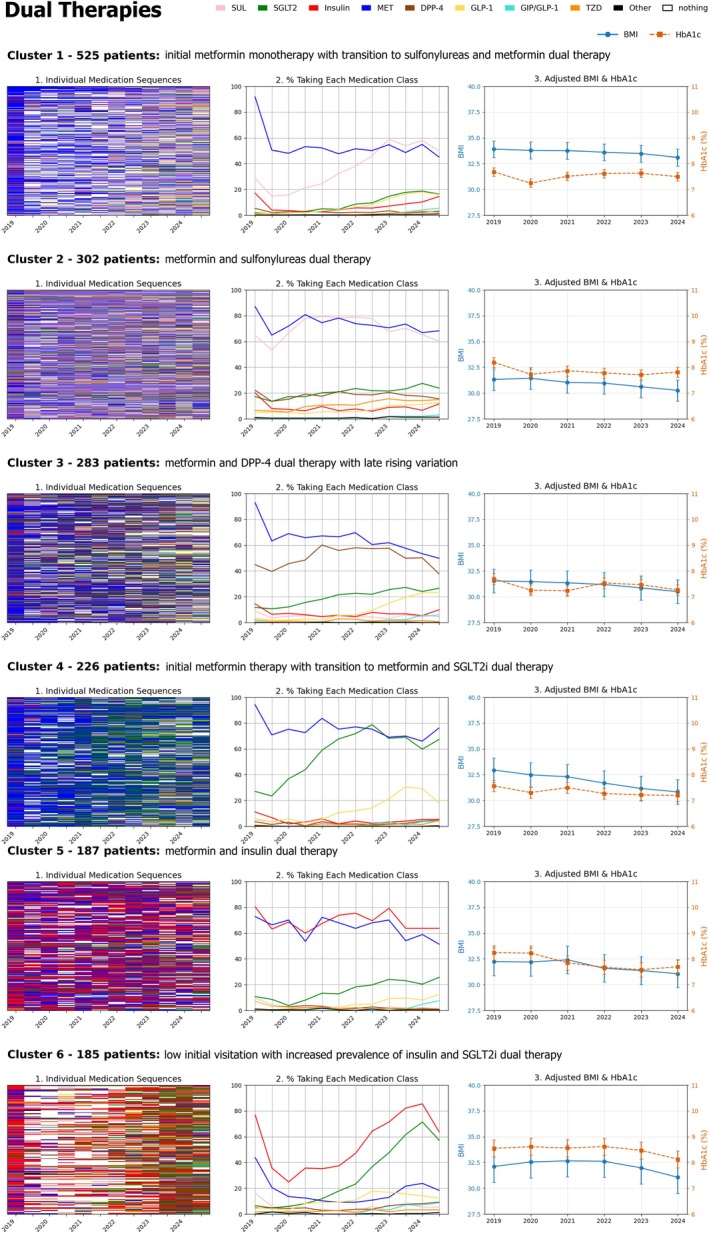
Dual‐therapy cluster visualisations. This figure presents three‐panel visualisations for six of eight clusters classified within the Dual Therapy group. The six clusters shown were chosen to span the distinct medication combinations and BMI/HbA1c trajectories within the group; the complete set is shown in Figure S10. Each panel includes (1) individual patient trajectory heatmaps, (2) medication usage distributions over time and (3) adjusted BMI and HbA1c trajectories where available.

**FIGURE 3:**
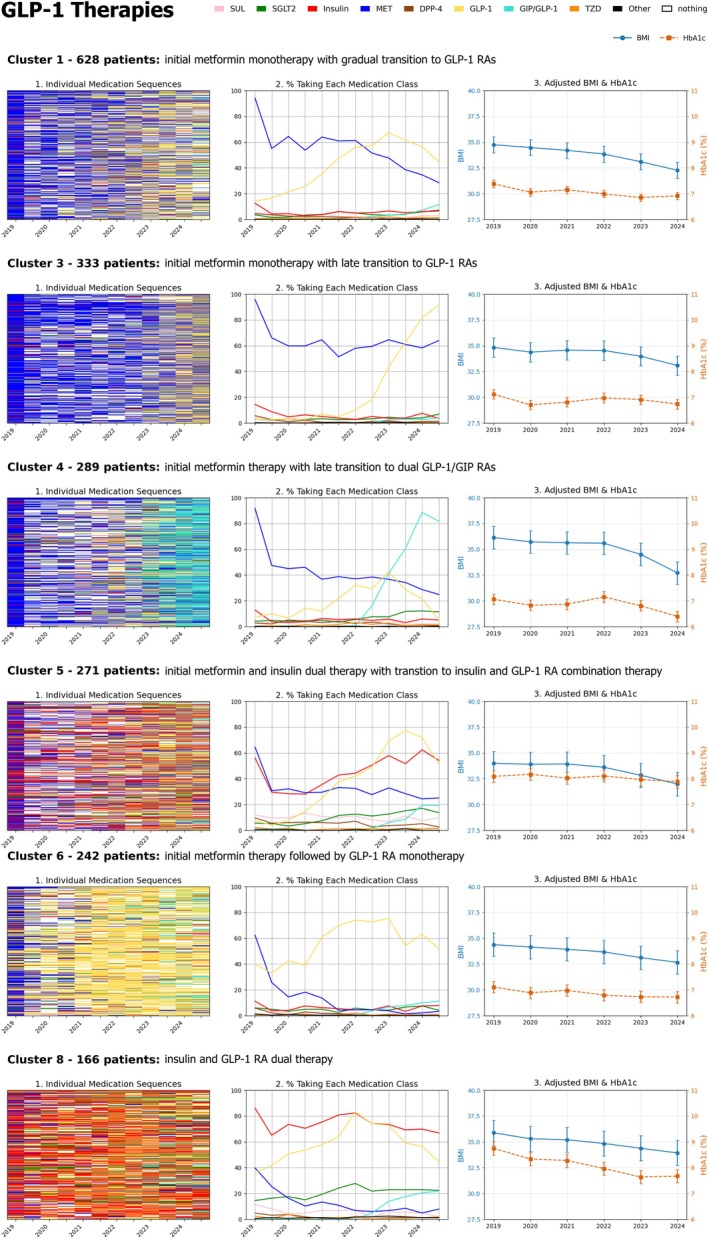
GLP‐1 RA–based therapy cluster visualisations. This figure presents three‐panel visualisations for six of 11 clusters classified within the GLP‐1 RA–Based Therapy group. The six clusters shown were chosen to span the distinct medication combinations and BMI/HbA1c trajectories within the group; the complete set is shown in Figures S12 and S13. Each panel includes (1) individual patient trajectory heatmaps, (2) medication usage distributions over time and (3) adjusted BMI and HbA1c trajectories where available.

During the first half of 2019, 6103 patients (65.4%) initiated monotherapy, 2174 (23.3%) dual therapy and 1050 (11.3%) complex therapy. Over follow‐up, 4833 patients (51.8%) were prescribed a GLP‐1 RA and 294 (13.8%) a dual GIP/GLP‐1 RA (Figures 1, 2, 3; Figures S10–S13). The distribution of patients across higher‐level prescribing trajectory categories by race/ethnicity and by HF/CKD status is shown in Figure S14.

Patient‐level comparisons of trajectory eligibility for HbA1c and BMI are provided in Tables S8 and S9, respectively. For BMI, missingness likely reflects heterogeneity in vital‐sign ascertainment across contributing TriNetX health systems and the BMI trajectory‐eligible subset had slightly higher prevalence in the Midwest region (Table S9). A last‐observation‐carried‐forward sensitivity analysis (Tables S10 and S11, Figure S15) produced cluster‐level trajectories concordant with the primary analysis.

### 3.2 Monotherapy Clusters

Monotherapy clusters included 2759 patients (29.6%), most of whom remained on single‐agent therapy throughout follow‐up (Figure 1). Clusters 1 and 2 show cyclic patterns that are likely a result of visitation frequency. Metformin monotherapy predominated (2533 patients), with clusters differing mainly by follow‐up intensity. Across metformin monotherapy clusters, adjusted BMI declined by 1.38 kg/m^2^ (95% CI, −1.60 to −1.16) and HbA1c by 0.16 percentage points (95% CI, −0.27 to −0.06) between 2019 and 2024 (Table S3). Insulin monotherapy (226 patients) was associated with greater comorbidity burden, including HF and CKD. Hispanic patients were overrepresented in the very low follow‐up cluster, Black patients in the metformin low follow‐up cluster and Asian patients in the metformin high follow‐up cluster (Table 2). Older adults (≥ 65 years) were more likely to belong to monotherapy trajectories (Table 3).

**TABLE 2:** Odds of membership in each cluster by race and ethnicity.

Cluster	#	*N*	Black, NH	Hispanic	Asian, NH	Other
Mono: MET (Low F/U)	1	765	1.43 (1.17–1.73); *p* ≤ 0.001***; pBH = 0.003**	1.07 (0.79–1.42); *p* = 0.654; pBH = 0.814	1.25 (0.92–1.66); *p* = 0.140; pBH = 0.281	1.38 (0.99–1.87); *p* = 0.047*; pBH = 0.123
Mono: MET (Low F/U)	2	707	1.14 (0.92–1.40); *p* = 0.227; pBH = 0.406	1.02 (0.76–1.35); *p* = 0.897; pBH = 0.935	1.20 (0.89–1.60); *p* = 0.224; pBH = 0.402	0.68 (0.44–1.00); *p* = 0.062; pBH = 0.147
Mono: MET (High F/U)	3	695	1.11 (0.89–1.37); *p* = 0.337; pBH = 0.528	1.06 (0.79–1.40); *p* = 0.666; pBH = 0.817	1.61 (1.21–2.12); *p* ≤ 0.001***; pBH = 0.004**	0.86 (0.58–1.23); *p* = 0.436; pBH = 0.639
Mono: MET (Very Low F/U)	4	363	1.03 (0.76–1.38); *p* = 0.856; pBH = 0.920	1.96 (1.43–2.66); *p* ≤ 0.001***; pBH ≤ 0.001***	1.04 (0.65–1.59); *p* = 0.861; pBH = 0.923	0.77 (0.43–1.27); *p* = 0.339; pBH = 0.529
Mono: INS	5	226	1.19 (0.83–1.68); *p* = 0.327; pBH = 0.519	0.79 (0.45–1.29); *p* = 0.377; pBH = 0.572	0.46 (0.21–0.87); *p* = 0.027*; pBH = 0.080	0.94 (0.50–1.63); *p* = 0.843; pBH = 0.914
Dual: MET + SUL	1	525	0.75 (0.57–0.98); *p* = 0.037*; pBH = 0.103	1.10 (0.81–1.47); *p* = 0.532; pBH = 0.725	0.68 (0.43–1.02); *p* = 0.078; pBH = 0.179	1.10 (0.75–1.56); *p* = 0.629; pBH = 0.796
Dual: MET + SUL	2	302	0.55 (0.35–0.82); *p* = 0.005**; pBH = 0.022*	1.66 (1.14–2.37); *p* = 0.007**; pBH = 0.025*	1.26 (0.79–1.91); *p* = 0.306; pBH = 0.496	1.89 (1.24–2.79); *p* = 0.002**; pBH = 0.010*
Dual: MET + DPP‐4i	3	283	0.89 (0.62–1.25); *p* = 0.504; pBH = 0.704	1.68 (1.14–2.42); *p* = 0.007**; pBH = 0.025*	1.45 (0.92–2.18); *p* = 0.094; pBH = 0.208	1.03 (0.58–1.71); *p* = 0.906; pBH = 0.936
Dual: MET + SGLT2i	4	226	0.54 (0.33–0.84); *p* = 0.010*; pBH = 0.033*	1.41 (0.91–2.13); *p* = 0.112; pBH = 0.240	2.36 (1.53–3.53); *p* ≤ 0.001***; pBH ≤ 0.001***	1.11 (0.59–1.92); *p* = 0.715; pBH = 0.845
Dual: MET + INS	5	187	1.35 (0.91–1.96); *p* = 0.125; pBH = 0.257	1.54 (0.96–2.40); *p* = 0.062; pBH = 0.147	0.93 (0.46–1.68); *p* = 0.817; pBH = 0.891	0.70 (0.29–1.41); *p* = 0.363; pBH = 0.560
Dual: INS + SGLT2i (Low F/U)	6	185	1.06 (0.71–1.54); *p* = 0.776; pBH = 0.880	0.70 (0.37–1.22); *p* = 0.236; pBH = 0.415	0.87 (0.45–1.54); *p* = 0.649; pBH = 0.811	0.52 (0.20–1.10); *p* = 0.123; pBH = 0.255
Dual: MET + TZD	7	126	0.84 (0.49–1.38); *p* = 0.516; pBH = 0.716	0.96 (0.46–1.79); *p* = 0.900; pBH = 0.936	0.92 (0.44–1.74); *p* = 0.817; pBH = 0.891	0.27 (0.04–0.87); *p* = 0.070; pBH = 0.161
Dual: MET + SUL (Declining F/U)	8	117	1.24 (0.75–1.97); *p* = 0.379; pBH = 0.573	0.86 (0.38–1.71); *p* = 0.691; pBH = 0.835	1.17 (0.54–2.27); *p* = 0.658; pBH = 0.815	0.48 (0.12–1.31); *p* = 0.219; pBH = 0.398
Complex: MET + SGLT2i + GLP‐1 RA	1	312	1.20 (0.87–1.62); *p* = 0.251; pBH = 0.433	1.36 (0.91–1.98); *p* = 0.119; pBH = 0.251	1.61 (1.03–2.42); *p* = 0.027*; pBH = 0.080	1.24 (0.73–1.98); *p* = 0.398; pBH = 0.595
Complex: MET + SUL + INS	2	153	0.78 (0.48–1.21); *p* = 0.286; pBH = 0.477	0.33 (0.13–0.69); *p* = 0.009**; pBH = 0.030*	0.81 (0.38–1.56); *p* = 0.567; pBH = 0.750	1.19 (0.61–2.10); *p* = 0.586; pBH = 0.768
Complex: INS + DPP‐4i + MET	3	62	1.20 (0.57–2.33); *p* = 0.614; pBH = 0.782	1.66 (0.70–3.52); *p* = 0.213; pBH = 0.388	1.08 (0.31–2.80); *p* = 0.890; pBH = 0.935	2.01 (0.75–4.55); *p* = 0.123; pBH = 0.255
Variant: INS ↔ MET	1	514	1.05 (0.82–1.32); *p* = 0.711; pBH = 0.844	0.69 (0.47–0.98); *p* = 0.046*; pBH = 0.121	0.43 (0.25–0.69); *p* = 0.001**; pBH = 0.006**	0.60 (0.36–0.94); *p* = 0.034*; pBH = 0.097
Variant: SUL‐based	2	390	0.99 (0.74–1.29); *p* = 0.922; pBH = 0.941	0.61 (0.38–0.91); *p* = 0.023*; pBH = 0.069	0.45 (0.24–0.78); *p* = 0.009**; pBH = 0.030*	1.34 (0.89–1.95); *p* = 0.147; pBH = 0.289
Variant: DPP‐4i‐based (Low F/U)	3	321	0.87 (0.63–1.19); *p* = 0.407; pBH = 0.603	0.61 (0.36–0.97); *p* = 0.050; pBH = 0.126	1.14 (0.74–1.70); *p* = 0.538; pBH = 0.730	1.43 (0.91–2.15); *p* = 0.102; pBH = 0.221
GLP‐1: MET → GLP‐1 RA (Early transition)	1	628	0.88 (0.71–1.09); *p* = 0.255; pBH = 0.437	0.57 (0.40–0.79); *p* = 0.001**; pBH = 0.005**	0.40 (0.23–0.63); *p* ≤ 0.001***; pBH = 0.002**	0.92 (0.63–1.30); *p* = 0.644; pBH = 0.810
GLP‐1: MET + INS → GLP‐1 RA (Low F/U)	2	424	1.18 (0.92–1.50); *p* = 0.191; pBH = 0.354	0.69 (0.47–0.99); *p* = 0.056; pBH = 0.138	0.58 (0.32–0.97); *p* = 0.053; pBH = 0.133	0.89 (0.55–1.36); *p* = 0.598; pBH = 0.775
GLP‐1: MET → GLP‐1 RA (Late transition)	3	333	1.09 (0.81–1.45); *p* = 0.565; pBH = 0.750	1.45 (1.01–2.03); *p* = 0.039*; pBH = 0.104	0.98 (0.57–1.59); *p* = 0.936; pBH = 0.949	1.20 (0.72–1.88); *p* = 0.458; pBH = 0.661
GLP‐1: MET → GIP/GLP‐1 RA	4	289	0.57 (0.40–0.79); *p* = 0.001**; pBH = 0.006**	0.54 (0.33–0.85); *p* = 0.011*; pBH = 0.037*	0.51 (0.25–0.92); *p* = 0.038*; pBH = 0.104	0.72 (0.39–1.21); *p* = 0.242; pBH = 0.423
GLP‐1: MET + INS → INS + GLP‐1 RA	5	271	0.96 (0.69–1.32); *p* = 0.797; pBH = 0.890	1.27 (0.86–1.83); *p* = 0.206; pBH = 0.376	0.34 (0.13–0.70); *p* = 0.009**; pBH = 0.033*	0.80 (0.43–1.37); *p* = 0.454; pBH = 0.661
GLP‐1: Early GLP‐1 RA	6	241	1.06 (0.76–1.45); *p* = 0.743; pBH = 0.862	0.47 (0.25–0.82); *p* = 0.014*; pBH = 0.045*	0.95 (0.52–1.62); *p* = 0.868; pBH = 0.925	1.04 (0.57–1.76); *p* = 0.894; pBH = 0.935
GLP‐1: SGLT2 → SGLT2 + GLP‐1 RA	7	187	1.02 (0.67–1.50); *p* = 0.932; pBH = 0.947	1.12 (0.65–1.83); *p* = 0.663; pBH = 0.816	1.90 (1.11–3.07); *p* = 0.013*; pBH = 0.042*	0.84 (0.37–1.64); *p* = 0.646; pBH = 0.810
GLP‐1: INS + GLP‐1 RA	8	166	0.93 (0.59–1.41); *p* = 0.729; pBH = 0.852	1.11 (0.65–1.81); *p* = 0.676; pBH = 0.821	1.42 (0.71–2.57); *p* = 0.285; pBH = 0.477	1.39 (0.72–2.47); *p* = 0.290; pBH = 0.481
GLP‐1: MET + INS → +GLP‐1 RA	9	143	1.36 (0.85–2.14); *p* = 0.188; pBH = 0.351	2.52 (1.58–3.94); *p* ≤ 0.001***; pBH ≤ 0.001***	1.70 (0.86–3.10); *p* = 0.099; pBH = 0.217	1.05 (0.43–2.15); *p* = 0.907; pBH = 0.936
GLP‐1: MET + SGLT2i + GLP‐1 RA	10	94	0.50 (0.23–0.98); *p* = 0.059; pBH = 0.143	0.80 (0.33–1.68); *p* = 0.589; pBH = 0.768	2.92 (1.56–5.16); *p* ≤ 0.001***; pBH = 0.003**	2.09 (0.99–3.99); *p* = 0.036*; pBH = 0.100
GLP‐1: MET + SUL → GLP‐1 RA	11	87	0.65 (0.33–1.17); *p* = 0.173; pBH = 0.329	0.57 (0.22–1.23); *p* = 0.192; pBH = 0.355	1.04 (0.40–2.28); *p* = 0.921; pBH = 0.941	0.53 (0.13–1.44); *p* = 0.282; pBH = 0.475

*Note:* White, Non‐Hispanic (NH) race/ethnicity served as the reference group. Values are expressed as odds ratios (ORs) with 95% confidence intervals (CIs); within each cell, *p* is the unadjusted *p*‐value from multivariable logistic regression and pBH is the Benjamini‐Hochberg false discovery rate‐adjusted *p*‐value, with significance stars (***p < 0.001, **p < 0.01, *p < 0.05) applied to each independently. Race categories reflect self‐reported race as recorded in the electronic health record. CI, confidence interval; DPP‐4i, dipeptidyl peptidase‐4 inhibitor; F/U, follow‐up; GIP/GLP‐1 RA, dual glucose‐dependent insulinotropic polypeptide and glucagon‐like peptide‐1 RA; GLP‐1 RA, glucagon‐like peptide‐1 RA; INS, insulin; MET, metformin; NH, non‐Hispanic; OR, odds ratio; SGLT2i, sodium‐glucose cotransporter‐2 inhibitor; SUL, sulfonylurea; TZD, thiazolidinedione.

**TABLE 3:** Odds of membership in each trajectory cluster by age category.

Group	#	*N*	55–64 years	≥ 65 years
Mono: MET (Low F/U)	1	765	1.21 (1.01–1.46); *p* = 0.042*; pBH = 0.111	1.54 (1.28–1.86); *p* ≤ 0.001***; pBH ≤ 0.001***
Mono: MET (Low F/U)	2	707	1.15 (0.95–1.39); *p* = 0.150; pBH = 0.292	1.40 (1.16–1.70); *p* ≤ 0.001***; pBH = 0.004**
Mono: MET (High F/U)	3	695	1.43 (1.18–1.73); *p* ≤ 0.001***; pBH = 0.002**	1.62 (1.33–1.97); *p* ≤ 0.001***; pBH ≤ 0.001***
Mono: MET (Very Low F/U)	4	363	1.18 (0.91–1.52); *p* = 0.222; pBH = 0.400	1.39 (1.07–1.81); *p* = 0.013*; pBH = 0.043*
Mono: INS	5	226	1.47 (1.05–2.09); *p* = 0.027*; pBH = 0.080	2.15 (1.54–3.02); *p* ≤ 0.001***; pBH ≤ 0.001***
Dual: MET + SUL	1	525	1.03 (0.84–1.27); *p* = 0.780; pBH = 0.881	0.95 (0.76–1.19); *p* = 0.670; pBH = 0.819
Dual: MET + SUL	2	302	1.60 (1.19–2.16); *p* = 0.002**; pBH = 0.010*	2.34 (1.75–3.13); *p* ≤ 0.001***; pBH ≤ 0.001***
Dual: MET + DPP‐4i	3	283	1.39 (1.04–1.84); *p* = 0.024*; pBH = 0.071	1.31 (0.96–1.77); *p* = 0.083; pBH = 0.188
Dual: MET + SGLT2i	4	226	0.94 (0.70–1.27); *p* = 0.710; pBH = 0.844	0.50 (0.33–0.72); *p* ≤ 0.001***; pBH = 0.003**
Dual: MET + INS	5	187	0.82 (0.56–1.19); *p* = 0.297; pBH = 0.489	1.49 (1.05–2.11); *p* = 0.023*; pBH = 0.070
Dual: INS + SGLT2i (Low F/U)	6	185	1.14 (0.79–1.62); *p* = 0.483; pBH = 0.682	1.25 (0.86–1.80); *p* = 0.235; pBH = 0.415
Dual: MET + TZD	7	126	1.31 (0.82–2.09); *p* = 0.262; pBH = 0.444	2.31 (1.50–3.60); *p* ≤ 0.001***; pBH = 0.001**
Dual: MET + SUL (Declining F/U)	8	117	1.30 (0.82–2.05); *p* = 0.257; pBH = 0.437	1.83 (1.16–2.89); *p* = 0.009**; pBH = 0.031*
Complex: MET + SGLT2i + GLP‐1 RA	1	312	1.01 (0.78–1.31); *p* = 0.925; pBH = 0.942	0.74 (0.54–1.00); *p* = 0.056; pBH = 0.138
Complex: MET + SUL + INS	2	153	1.11 (0.76–1.61); *p* = 0.588; pBH = 0.768	0.93 (0.61–1.41); *p* = 0.743; pBH = 0.862
Complex: INS + DPP‐4i + MET	3	62	1.14 (0.59–2.18); *p* = 0.686; pBH = 0.831	1.92 (1.05–3.55); *p* = 0.035*; pBH = 0.098
Variant: INS ↔ MET	1	514	1.03 (0.83–1.28); *p* = 0.789; pBH = 0.886	1.28 (1.03–1.60); *p* = 0.028*; pBH = 0.081
Variant: SUL‐based	2	390	1.07 (0.83–1.37); *p* = 0.609; pBH = 0.779	1.45 (1.13–1.87); *p* = 0.003**; pBH = 0.015*
Variant: DPP‐4i‐based (Low F/U)	3	321	1.44 (1.09–1.91); *p* = 0.011*; pBH = 0.037*	1.62 (1.22–2.16); *p* ≤ 0.001***; pBH = 0.005**
GLP‐1: MET → GLP‐1 RA (Early)	1	628	0.68 (0.56–0.82); *p* ≤ 0.001***; pBH ≤ 0.001***	0.38 (0.30–0.49); *p* ≤ 0.001***; pBH ≤ 0.001***
GLP‐1: MET + INS → GLP‐1 RA	2	424	0.72 (0.57–0.90); *p* = 0.004**; pBH = 0.019*	0.57 (0.43–0.73); *p* ≤ 0.001***; pBH ≤ 0.001***
GLP‐1: MET→GLP‐1 RA (Late)	3	333	0.82 (0.64–1.05); *p* = 0.113; pBH = 0.240	0.38 (0.27–0.53); *p* ≤ 0.001***; pBH ≤ 0.001***
GLP‐1: MET→GIP/GLP‐1 RA	4	289	0.58 (0.44–0.76); *p* ≤ 0.001***; pBH ≤ 0.001***	0.25 (0.17–0.36); *p* ≤ 0.001***; pBH ≤ 0.001***
GLP‐1: MET + INS → INS + GLP‐1 RA	5	271	0.75 (0.57–0.99); *p* = 0.048*; pBH = 0.125	0.53 (0.38–0.74); *p* ≤ 0.001***; pBH = 0.002**
GLP‐1: Early GLP‐1 RA	6	241	0.63 (0.46–0.84); *p* = 0.002**; pBH = 0.011*	0.41 (0.28–0.59); *p* ≤ 0.001***; pBH ≤ 0.001***
GLP‐1: SGLT2 → SGLT2 + GLP‐1 RA	7	187	1.13 (0.82–1.55); *p* = 0.464; pBH = 0.668	0.55 (0.35–0.83); *p* = 0.006**; pBH = 0.023*
GLP‐1: INS + GLP‐1 RA	8	166	0.96 (0.67–1.36); *p* = 0.812; pBH = 0.891	0.74 (0.48–1.12); *p* = 0.164; pBH = 0.313
GLP‐1: MET + INS → +GLP‐1 RA	9	143	0.87 (0.58–1.28); *p* = 0.485; pBH = 0.683	0.85 (0.54–1.30); *p* = 0.456; pBH = 0.661
GLP‐1: MET + SGLT2i + GLP‐1 RA	10	94	0.62 (0.38–0.99); *p* = 0.048*; pBH = 0.125	0.27 (0.13–0.51); *p* ≤ 0.001***; pBH = 0.001**
GLP‐1: MET + SUL → GLP‐1 RA	11	87	0.78 (0.47–1.25); *p* = 0.301; pBH = 0.490	0.49 (0.26–0.88); *p* = 0.022*; pBH = 0.068

*Note:* Age < 55 years served as the reference group. Values are expressed as odds ratios (ORs) with 95% confidence intervals (CIs); within each cell, *p* is the unadjusted *p*‐value from multivariable logistic regression and pBH is the Benjamini–Hochberg false discovery rate‐adjusted *p*‐value, with significance stars (***p < 0.001, **p < 0.01, *p < 0.05) applied to each independently. Glucagon‐like peptide‐1 (GLP‐1) therapy is defined as a treatment pattern that includes a GLP‐1 RA (RA) component. Variant therapy denotes treatment patterns that do not align with any other predefined therapy group. CI, confidence interval; DPP‐4i, dipeptidyl peptidase‐4 inhibitor; F/U, follow‐up; GIP/GLP‐1 RA, dual glucose‐dependent insulinotropic polypeptide and glucagon‐like peptide‐1 RA; GLP‐1 RA, glucagon‐like peptide‐1 RA; INS, insulin; MET, metformin; NH, non‐Hispanic; OR, odds ratio; SGLT2i, sodium‐glucose cotransporter‐2 inhibitor; SUL, sulfonylurea; TZD, thiazolidinedione.

### 3.3 Dual‐Therapy Clusters

Dual‐therapy clusters included 1951 patients (20.9%) who generally maintained stable two‐drug regimens (Figure 2 **;** Figure S10). Adjusted BMI reductions ranged from −0.89 kg/m^2^ (95% CI, −1.27 to −0.51) for metformin plus sulfonylurea to −2.12 kg/m^2^ (95% CI, −2.84 to −1.40) for metformin plus SGLT2 inhibitors (Table S3). Most dual‐therapy clusters also showed modest HbA1c reductions. Black patients had lower odds of membership in metformin plus sulfonylurea and metformin plus SGLT2 inhibitor clusters, whereas Asian patients were overrepresented in the metformin plus SGLT2 inhibitor cluster. Older age was associated with sulfonylurea‐ and TZD‐based therapies and with lower use of metformin plus SGLT2 inhibitors.

### 3.4 Complex‐Therapy Clusters

Complex‐therapy clusters included 527 patients (5.7%) (Figure S11). The combination of metformin, SGLT2 inhibitor and GLP‐1 RA was associated with significant BMI reduction (−1.63 kg/m^2^; 95% CI, −2.28 to −0.98), whereas HbA1c changes were modest (Table S3). Other insulin‐based triple therapies showed minimal or non‐significant changes in BMI and HbA1c. Demographic differences were limited, although Hispanic patients had lower odds of membership in insulin‐based triple‐therapy clusters.

### 3.5 Variant‐Therapy Clusters

Variant‐therapy clusters included 1225 patients (13.0%) with prescribing patterns outside standard therapeutic categories (Figure S11). The largest cluster (514 patients) involved alternating insulin and metformin use and was associated with reductions in BMI (−1.68 kg/m^2^; 95% CI, −2.23 to −1.12) and HbA1c (−0.80 percentage points; 95% CI, −1.10 to −0.50) (Table S3). Other variant clusters showed modest BMI reductions and minimal HbA1c changes. Asian patients had lower odds of membership, while variant therapies were more common among older adults.

### 3.6 GLP‐1 RA‐Based Therapy Clusters

GLP‐1 RA‐based therapy clusters included 2865 patients (30.7%) (Figure 3; Figures S12 and S13). These clusters generally had higher baseline BMI and greater BMI reductions during follow‐up. The most common trajectory involved transition from metformin to GLP‐1 RA therapy (961 patients [10.3%]) and was associated with reductions in BMI (−2.20 kg/m^2^; 95% CI, −2.55 to −1.85) and HbA1c (−0.44 percentage points; 95% CI, −0.60 to −0.27) (Table S3). Transition from metformin to dual GIP/GLP‐1 RA therapy had large reductions in BMI (−3.43 kg/m^2^; 95% CI, −4.08 to −2.78) and HbA1c (−0.67 percentage points; 95% CI, −0.98 to −0.36).

Substantial heterogeneity in GLP‐1 RA trajectory membership was observed by race/ethnicity and age. Hispanic patients had lower odds of membership in early GLP‐1 RA transition clusters, while Asian patients were more likely to belong to SGLT2 inhibitor–based or more complex GLP‐1 RA regimens. Older adults, particularly those aged ≥ 65 years, had lower odds of membership across most GLP‐1 RA trajectories.

## 4 Discussion

In this longitudinal EHR study, we used data‐driven trajectory clustering to characterise real‐world diabetes pharmacotherapy over time. Compared with national CDC estimates of U.S. adults with diagnosed diabetes, the cohort showed comparable sex and Black, Non‐Hispanic distributions, modestly higher White, Non‐Hispanic and Asian, Non‐Hispanic representation, lower Hispanic/Latinx representation and a younger overall age distribution [1]. The approach identified 30 distinct prescribing trajectories spanning monotherapy, dual therapy, complex regimens, variant patterns and GLP‐1 RA‐based pathways. By preserving temporal ordering without relying on predefined treatment categories, this framework captured treatment escalation, switching, de‐escalation and early care disengagement patterns that are difficult to identify using cross‐sectional analyses. Beyond descriptive epidemiology, these longitudinal prescribing trajectories may provide an important foundation for future machine learning research using national EHR networks by enabling development of temporally informed models for treatment prediction, treatment‐effect heterogeneity, risk stratification and learning health system applications [24].

In the United States, coverage for higher‐cost glucose‐lowering therapies is fragmented across payers without a universal reimbursement framework and pharmacy dispensing or claims data would be required to directly assess medication fills [25, 26, 27]. In this study, substantial heterogeneity was observed in the timing and adoption of GLP‐1 RA therapies. GLP‐1 RA‐based trajectories represented one of the largest treatment groups, reflecting increasing uptake of incretin‐based therapies during the study period. Most GLP‐1 RAs were introduced after initial therapy because they only became widely available after 2022 and initiation timing varied substantially across clusters, consistent with evolving evidence, regulatory approvals and insurance coverage [28, 29]. These findings suggest that the adoption of newer therapies may lag guideline recommendations for earlier use in appropriate patients.

Longitudinal BMI and HbA1c analyses provided clinical context beyond descriptive clustering and demonstrated substantial heterogeneity in metabolic outcomes, even within the same medication classes consistent with prior studies [30, 31]. These findings should be interpreted as descriptive associations rather than treatment effects because clusters differed in baseline BMI, age, comorbidity burden and visitation intensity. GLP‐1 RA–based trajectories generally showed the largest reductions in BMI and HbA1c. More complex insulin‐intensive trajectories showed smaller metabolic improvements, likely reflecting greater disease severity and treatment constraints.

Prescribing trajectory membership varied by race and ethnicity, particularly among monotherapies and GLP‐1 RA‐based therapies. Hispanic patients had lower odds of membership in early GLP‐1 RA transition clusters, while Asian patients showed differential representation across regimen complexity. These findings suggest that disparities are most apparent in pathways involving treatment intensification and newer therapies, consistent with prior observational studies [8, 32, 33, 34]. Because TriNetX lacks detailed socioeconomic and pharmacy claims data, the relative contributions of insurance design, out‐of‐pocket costs, language access and provider‐level factors could not be assessed directly. In future work, we plan to incorporate additional datasets with more detailed individual‐ and address‐level social determinants of health (SDOH) information, such as Epic Systems Cosmos, which may improve characterisation of socioeconomic and geographic influences on prescribing trajectories [35].

Age was independently associated with diabetes prescribing trajectory membership. Older adults, particularly those aged 65 years or older, were more often represented in monotherapy and lower‐engagement trajectories and consistently showed lower odds of membership in GLP‐1 RA–based clusters. Consistent with prior research, GLP‐1 RA use in this cohort was concentrated among younger adults with higher BMI [36]. These findings suggest substantial age‐related differences in observed treatment pathways. This pattern is clinically relevant given the lower hypoglycemia risk and potential cardiovascular benefit associated with newer glucose‐lowering agents and aligns with prior evidence of therapeutic inertia and continued reliance on insulin or sulfonylureas among older adults [36, 37]. While clinical considerations such as comorbidity burden and polypharmacy may partially explain these patterns, the observed differences may also reflect factors not captured in this dataset, including insurance design, patient preference and provider specialty. Given prior evidence that hypoglycemia disproportionately affects Non‐White populations [38, 39], the lower odds of older adult membership in GLP‐1 RA–based clusters observed here may further compound existing disparities. Together, these findings highlight the value of longitudinal outcome assessment for interpreting real‐world treatment pathways.

## 5 Limitations

This study has several limitations. The cohort was restricted to patients with longitudinal care within the TriNetX network, which may limit generalisability and introduce selection bias. Although the EHR‐based phenotype likely improved identification of incident T2D compared with claims‐based approaches, some misclassification remains probable because undiagnosed or uncaptured disease may not appear in the EHR record. Additionally, TriNetX populations may differ from the broader U.S. population with diabetes in age, racial/ethnic composition and socioeconomic status, potentially limiting external validity. Also, interpretation of GLP‐1 RA trajectories is limited by shorter follow‐up because these therapies entered routine clinical practice later in the study period.

Patient‐level comparisons across clusters should also be interpreted cautiously because baseline characteristics differed substantially between groups. Although multivariable models adjusted for measured covariates, residual confounding from unmeasured factors (e.g., insurance status, pharmacy dispensing data, medication adherence, out‐of‐pocket costs, prior authorisation requirements) remains likely. Patients in insulin‐intensive and more complex trajectories had higher rates of HF and CKD, suggesting that cluster membership may partly reflect underlying disease severity and treatment constraints rather than prescribing preference alone. Because these conditions are associated with both SGLT2 inhibitor and GLP‐1 RA use, they could influence observed differences in BMI and HbA1c trajectories despite adjustment for measured comorbidities.

Finally, methodological choices, including 6‐month interval aggregation and binary drug‐class encoding, may have obscured shorter‐term medication changes such as dose titration, temporary discontinuation, or intermittent use. However, the sensitivity analyses showed that study findings are robust across these methodological choices. As an observational study, findings represent associations rather than causal effects.

## 6 Conclusion

In adults with T2D, clustering longitudinal EHR data identified multiple distinct prescribing trajectories that reflect substantial heterogeneity in real‐world treatment pathways. Notably, differences in trajectory membership by age and race/ethnicity were observed, particularly in the adoption and timing of GLP‐1‐based therapies. Beyond these descriptive findings, this framework may support future studies linking treatment trajectories to long‐term outcomes and inform characterisation of variation in real‐world diabetes care. For clinical practice, this approach could be adapted by individual health systems to benchmark local prescribing patterns against the national trajectories described here, identify clusters consistent with therapeutic inertia and monitor guideline‐concordant adoption of newer therapies over time.

## Author Contributions

**Tobias S. Lux:** conceptualisation, data curation, formal analysis, investigation, writing – original draft, writing – review and editing. **Dongkun Lee:** conceptualisation, data curation, formal analysis, investigation, writing – original draft, writing – review and editing. **Jeff M. Phillips:** conceptualisation, funding acquisition, investigation, methodology, supervision, writing – original draft, writing – review and editing. **Julio C. Facelli:** conceptualisation, investigation, methodology, writing – original draft, writing – review and editing. **Daniel C. Malone:** conceptualisation, investigation, supervision, writing – original draft, writing – review and editing. **Matthew Wahl:** conceptualisation, funding acquisition, investigation, writing – original draft, writing – review and editing. **Deepika Reddy:** conceptualisation, investigation, writing – original draft, writing – review and editing. **Farrant Sakaguchi:** investigation, writing – original draft, writing – review and editing. **Ramkiran Gouripeddi:** investigation, methodology, writing – original draft, writing – review and editing. **Alexander S. Millar:** data curation, investigation, writing – original draft, writing – review and editing. **Matthew J. O'Brien:** investigation, writing – original draft, writing – review and editing. **Joshua S. Choi:** investigation, writing – review and editing. **Jittrapol Sutejitsiri:** investigation, writing – original draft, writing – review and editing. **Kensaku Kawamoto:** investigation, supervision, writing – original draft, writing – review and editing. **Polina V. Kukhareva:** conceptualisation, data curation, formal analysis, funding acquisition, investigation, methodology, project administration, resources, software, supervision, validation, visualisation, writing – original draft, writing – review and editing.

## Funding

Polina V. Kukhareva was supported by the NIDDK under award number K01DK142045. Tobias S. Lux and Dongkun Lee were supported by the University of Utah Data Exploration and Learning for Precision Health Intelligence (DELPHI) Data Science Initiative Seed Grant, *Decoding Diabetes Treatment Pathways: AI‐Driven Insights from Real‐World Data* (Multiple Principal Investigators: Polina V. Kukhareva, Matthew Wahl and Jeff M. Phillips). Joshua S. Choi was supported by the National Library of Medicine under grant number T15LM007124. Matthew J. O'Brien was supported by the National Institute of Diabetes and Digestive and Kidney Diseases (NIDDK) under award number P30DK092949. Alexander S. Millar was supported by the Department of Defence (DoD) SMART Scholarship‐for‐Service Program. The sponsors had no role in the design or conduct of the study; the collection, management, analysis, or interpretation of the data; the preparation, review, or approval of the manuscript; or the decision to submit it for publication. The content is solely the responsibility of the authors and does not necessarily represent the official views of the National Institutes of Health or other funding organisations.

## Conflicts of Interest

During the 36 months prior to publication, Kensaku Kawamoto reports honoraria, consulting, sponsored research, writing assistance, licencing, or co‐development outside of the submitted work with Hitachi, Pfizer, RTI International, NORC at the University of Chicago, the University of Pennsylvania, the University of Colorado, the University of Michigan, Yale University, Elsevier, Beckman Coulter, Surescripts, MD Aware, Custom Clinical Decision Support and the U.S. Office of the National Coordinator for Health IT (via Security Risk Solutions) in the area of health information technology. He was also an unpaid board member of the non‐profit Health Level Seven International health IT standard development organisation, he was an unpaid member of the U.S. Health Information Technology Advisory Committee and he has helped develop a number of health IT tools which may be commercialised to enable wider impact. Polina V. Kukhareva reports consulting with NORC at the University of Chicago; and Daniel C. Malone reports consulting with Gilead, Sanofi, Tandem, Sarepta, Biogen, InflaRx and Humacyte. None of these relationships have direct relevance to the manuscript but are reported in the interest of full disclosure. The remaining authors declare no conflicts of interest.

## Supporting information


**Table S1:** Clinical Codes Used in the Study.
**Table S2:** Primary Analytic Cohort vs. Early Disengagement Clusters.
**Table S3:** Practical Summary of Higher‐Level Prescribing Trajectory Categories.
**Table S4:** Most Prevalent Prescriptions and Comorbidities in Each Cluster.
**Table S5:** Adjusted Odds Ratios for HF/CKD by Cluster.
**Table S6:** Patient counts used for adjusted BMI and HbA1c trajectories.
**Table S7:** Patient Characteristics by Race/Ethnicity.
**Table S8:** HbA1c Trajectory‐Eligible vs. Ineligible Patients.
**Table S9:** BMI Trajectory‐Eligible vs. Ineligible Patients.
**Table S10:** Cluster‐level LOCF sensitivity analysis for HbA1c trajectories.
**Table S11:** Cluster‐level LOCF sensitivity analysis for BMI trajectories.
**Figure S1:** Cohort Construction and Patient
Selection Flow diagram showing inclusion and exclusion criteria used to derive the final study cohort. Adults (≥ 18 years) with type 2 diabetes (T2D) who were alive in 2019 and had at least one glucose‐lowering medication prescription were identified. Patients were sequentially excluded based on encounter history, medication prescriptions, diagnosis timing, prior glucose‐lowering therapy, evidence of uncontrolled hyperglycaemia, pregnancy‐related diabetes (ICD‐10 O24*) and residence outside the United States or unknown location. H1 and H2 denote the first (January–June) and second (July–December) halves of a calendar year, respectively
**Figure S2:** Early Disengagement Cluster Visualisations.
This eFigure presents two‐panel cluster visualisations for patients classified as external to the primary analytic cohort. Early disengagement patients were identified as those assigned to clusters in which more than 70% of patients had medication data concentrated in the first 1–2 study intervals with minimal subsequent prescribing activity, consistent with care occurring substantially outside of TriNetX health systems. Each cluster visualisation includes (1) individual prescribing trajectory heatmaps and (2) medication class prescribing over time. Because sustained longitudinal observation is required for trajectory analysis, these clusters were analysed separately and are presented here to characterise distinct patterns of limited healthcare engagement. Abbreviations: CI, confidence interval; DPP‐4i, dipeptidyl peptidase‐4 inhibitor; F/U, follow‐up; GIP/GLP‐1 RA, dual glucose‐dependent insulinotropic polypeptide and glucagon‐like peptide‐1 RA; GLP‐1 RA, glucagon‐like peptide‐1 RA; INS, insulin; MET, metformin; NH, non‐Hispanic; OR, odds ratio; SGLT2i, sodium‐glucose cotransporter‐2 inhibitor; SUL, sulfonylurea; TZD, thiazolidinedione.
**Figure S3:** Sensitivity Analysis: Therapy Group Concordance Under Alternative Filtering Approach.
This eFigure presents the concordance between original therapy group assignments (post hoc cluster‐level filtering) and therapy group assignments derived from a sensitivity analysis using patient‐level sparsity filtering prior to re‐clustering. The heatmap displays row‐normalised percentages: each cell shows the proportion of patients from a given original therapy group (rows) assigned to each sensitivity analysis group (columns). Diagonal values represent the concordance rate (the percentage of patients retaining the same therapy group classification). Re‐clustering was performed using Ward's linkage with *k* = 40 on patients with ≤ 70% empty medication bins. Each new cluster's medication profile (proportion of patients on each drug class per time bin) was compared to the 30 original cluster profiles using cosine similarity and assigned to the therapy group of its closest match.
**Figure S4:** Sensitivity Analysis: Therapy Group Concordance Under 3‐Month Binning.
Concordance between original therapy group assignments (computed using 6‐month bins) and therapy group assignments derived from re‐clustering after rebuilding patient trajectories at 3‐month resolution (24 quarterly bins, 2019–2024). The heatmap displays row‐normalised percentages: each cell shows the proportion of patients from a given original therapy group (rows) assigned to each 3‐month‐binning therapy group (columns). Diagonal values represent concordance (the percentage of patients retaining the same therapy group classification). Re‐clustering was performed using Ward's linkage with squared Euclidean distance on the full cohort (*n* = 18 652) at *k* = 40. Medication profiles for both new and original clusters were computed in the shared 6‐month feature space (proportion of patients on each drug class per six‐month bin); each new cluster was matched to the original cluster with the highest cosine similarity and inherited that cluster's therapy group label. Unlike the patient‐level sparsity sensitivity analysis (eFigure 3), the Early Dropout group is included here because no patient‐level sparsity filter was applied prior to re‐clustering.
**Figure S5:** Sensitivity Analysis: Therapy Group Concordance Under Prescription‐Time Alignment.
Concordance between original therapy group assignments (computed from trajectories anchored to January 2019) and therapy group assignments derived from re‐clustering after rebuilding patient trajectories anchored to each patient's individual first glucose‐lowering medication prescription date. The heatmap displays row‐normalised percentages: each cell shows the proportion of patients from a given original (calendar‐time) therapy group (rows) assigned to each prescription‐time therapy group (columns). Diagonal values represent concordance (the percentage of patients retaining the same therapy group classification); row and column ordering is determined by descending diagonal concordance. Re‐clustering used Ward's linkage with squared Euclidean distance on the full cohort (*n* = 18 652) at *k* = 40. Trajectories were constructed using 11 six‐month bins anchored at the patient's first prescription date (99‐dimensional feature space) so that every patient contributed the same number of complete bins. Each new cluster was matched to the original cluster with the highest cosine similarity of medication profiles in the shared 99‐dimensional feature space (11 six‐month bins × 9 drug classes); original cluster centroids were truncated from 12 bins to 11 to occupy the same feature space as the prescription‐time centroids. As in eFigure 4, the Early Dropout group is included because no patient‐level sparsity filter was applied prior to re‐clustering.
**Figure S6:** Vectorised Patient Prescribing Trajectory.
Schematic illustrating transformation of patient‐level prescription histories into a binary feature matrix. Prescription events are aggregated into half‐year intervals (H1: January–June; H2: July–December). Each row corresponds to a time interval and each column to a medication class, with matrix entries $M_{\mathrm{ij}}=1$indicating that medication jwas prescribed during interval i and 0 otherwise. The resulting matrix is flattened into a fixed‐length binary vector for clustering.
**Figure S7:** Comparison of Agglomerative Linkage Methods at *k* = 40.
Each panel applies a different classical agglomerative linkage method to the same 18 652‐patient input matrix using Euclidean distance: (A) single linkage, (B) complete linkage, (C) average linkage and (D) Ward's linkage. Heatmap conventions match eFigure 9.
**Figure S8:** Internal Validity Diagnostics for Selecting the Number of Clusters (*k*).
Two complementary criteria for choosing k are presented across *k* = 2 to 50, computed using Ward's linkage with squared Euclidean distance on the full clustering input matrix (*n* = 18 652 patients × 108‐dimensional prescribing trajectory vectors). (A) Within‐cluster sum of squares (WCSS), used in the elbow method. (B) Gap statistic, computed against B = 10 uniform‐random reference. The selected value *k* = 40 is marked in both panels.
**Figure S9:** Patient‐Level Prescribing Trajectory Heatmaps Across Increasing Values of *k*.
Each panel shows the same 18 652 patients clustered with Ward's linkage and squared Euclidean distance at *k* = 20, 30, 40 and 50. Each row of each heatmap is one patient; columns are six‐month bins from 2019‐H1 through 2024‐H2; colours encode medication class as shown in the legend. Black horizontal lines separate clusters. Abbreviations: DPP‐4i, dipeptidyl peptidase‐4 inhibitor; GIP/GLP‐1 RA, dual glucose‐dependent insulinotropic polypeptide and glucagon‐like peptide‐1 RA; GLP‐1 RA, glucagon‐like peptide‐1 RA; INS, insulin; MET, metformin; SGLT2i, sodium‐glucose cotransporter‐2 inhibitor; SUL, sulfonylurea; TZD, thiazolidinedione.
**Figure S10:** Comprehensive Dual Therapy Cluster Visualisations.
This eFigure presents three‐panel visualisations for all clusters classified within the Dual Therapy group. Each panel includes [1] individual patient trajectory heatmaps, [2] medication prescribing distributions over time and [3] adjusted BMI and HbA1c trajectories where available. A subset of representative clusters from this group is displayed in the main manuscript figures, while this eFigure provides visualisations for all clusters in this group. Abbreviations: CI, confidence interval; DPP‐4i, dipeptidyl peptidase‐4 inhibitor; F/U, follow‐up; GIP/GLP‐1 RA, dual glucose‐dependent insulinotropic polypeptide and glucagon‐like peptide‐1 RA; GLP‐1 RA, glucagon‐like peptide‐1 RA; INS, insulin; MET, metformin; NH, non‐Hispanic; OR, odds ratio; SGLT2i, sodium‐glucose cotransporter‐2 inhibitor; SUL, sulfonylurea; TZD, thiazolidinedione.
**Figure S11:** Complex and Variant Therapy Cluster Visualisations.
This figure presents three‐panel visualisations for all clusters classified within the Complex and Variant therapy groups. Each panel includes (1) individual patient trajectory heatmaps, (2) medication usage distributions over time and (3) adjusted BMI and HbA1c trajectories where available. Abbreviations: CI, confidence interval; DPP‐4i, dipeptidyl peptidase‐4 inhibitor; F/U, follow‐up; GIP/GLP‐1 RA, dual glucose‐dependent insulinotropic polypeptide and glucagon‐like peptide‐1 RA; GLP‐1 RA, glucagon‐like peptide‐1 RA; INS, insulin; MET, metformin; NH, non‐Hispanic; OR, odds ratio; SGLT2i, sodium‐glucose cotransporter‐2 inhibitor; SUL, sulfonylurea; TZD, thiazolidinedione.
**Figure S12:** Comprehensive GLP‐1 Therapy Cluster Visualisations (clusters 1–6).
This eFigure presents three‐panel visualisations for clusters 1–6 within the GLP‐1 therapy group. Each panel includes (1) individual patient trajectory heatmaps, (2) medication usage distributions over time and (3) adjusted BMI and HbA1c trajectories, where available. Representative clusters from this group are shown in the main manuscript figures, while this eFigure provides comprehensive visualisations for the first six clusters to ensure transparency of cluster‐level patterns. Abbreviations: CI, confidence interval; DPP‐4i, dipeptidyl peptidase‐4 inhibitor; F/U, follow‐up; GIP/GLP‐1 RA, dual glucose‐dependent insulinotropic polypeptide and glucagon‐like peptide‐1 RA; GLP‐1 RA, glucagon‐like peptide‐1 RA; INS, insulin; MET, metformin; NH, non‐Hispanic; OR, odds ratio; SGLT2i, sodium‐glucose cotransporter‐2 inhibitor; SUL, sulfonylurea; TZD, thiazolidinedione.
**Figure S13:** Comprehensive GLP‐1 Therapy Cluster Visualisations (clusters 7–11).
This eFigure presents three‐panel visualisations for clusters 7–11 within the GLP‐1 therapy group. Each panel includes (1) individual patient trajectory heatmaps, (2) medication usage distributions over time and (3) adjusted BMI and HbA1c trajectories, where available. Representative clusters from this group are shown in the main manuscript figures, while this eFigure provides comprehensive visualisations for the last five clusters to ensure transparency of cluster‐level patterns. Abbreviations: CI, confidence interval; DPP‐4i, dipeptidyl peptidase‐4 inhibitor; F/U, follow‐up; GIP/GLP‐1 RA, dual glucose‐dependent insulinotropic polypeptide and glucagon‐like peptide‐1 RA; GLP‐1 RA, glucagon‐like peptide‐1 RA; INS, insulin; MET, metformin; NH, non‐Hispanic; OR, odds ratio; SGLT2i, sodium‐glucose cotransporter‐2 inhibitor; SUL, sulfonylurea; TZD, thiazolidinedione.
**Figure S14:** Distribution of Higher‐Level Prescribing Trajectory Categories by Race/Ethnicity and by HF/CKD Status. Each bar shows the percentage of patients within a given stratum (race/ethnicity in Panel A; composite HF/CKD status in Panel B) assigned to each of the five higher‐level prescribing trajectory categories defined in eTable 3 (Monotherapy, Dual Therapy, Complex Therapy, Variant Therapy, GLP‐1 Therapy). Bars sum to 100% within each stratum; stratum‐level denominators are shown beneath each x‐axis label. The composite HF/CKD indicator follows the same definition used elsewhere in the analysis (documented heart failure or chronic kidney disease between January 1 and June 1, 2019). The figure reflects the primary analytic cohort; early‐disengagement clusters are analysed separately (eFigure 2).
**Figure S15:** Sensitivity Analysis: BMI and HbA1c Trajectories Under LOCF Imputation.
Overlay of original (complete‐case) and LOCF‐imputed adjusted BMI and HbA1c trajectories for representative clusters spanning the five therapy groups (Monotherapy—Cluster 1, Dual Therapy—Cluster 1, Complex Therapy—Cluster 1, Variant Therapy—Cluster 1, GLP‐1 Therapy—Cluster 1). Solid blue lines show the original analysis; dashed orange lines show the LOCF sensitivity analysis. Visual concordance between the two trajectories supports robustness of the manuscript's primary findings to relaxed measurement‐completeness requirements.
**Figure S16:** Cluster‐Level Patient Characteristics—Standardised View.
This eFigure presents the manuscript Table 1 characteristics for each of the 30 primary clusters as a heatmap. Rows are clusters, ordered by therapy group to match manuscript Tables 2 and 3, with horizontal lines demarcating group boundaries. Columns are mean age, % female, the racial/ethnic distribution, mean baseline HbA1c and BMI and the prevalence of documented HF and CKD. Each cell is annotated with the raw value and shaded by the per‐column z‐score across the 30 clusters: red indicates values above the cluster‐level mean, blue indicates values below and saturation reflects the absolute z‐score on a shared scale centred at zero. Standardisation is applied per column so that variables on different native scales remain visually comparable. Abbreviations: BMI, body mass index; CKD, chronic kidney disease; DPP‐4i, dipeptidyl peptidase‐4 inhibitor; F/U, follow‐up; GIP/GLP‐1 RA, dual glucose‐dependent insulinotropic polypeptide and glucagon‐like peptide‐1 RA; GLP‐1 RA, glucagon‐like peptide‐1 RA; HbA1c, glycated haemoglobin; HF, heart failure; INS, insulin; MET, metformin; NH, non‐Hispanic; SGLT2i, sodium‐glucose cotransporter‐2 inhibitor; SUL, sulfonylurea; TZD, thiazolidinedione.

## Data Availability

The data underlying this article cannot be shared publicly due to TriNetX policies.

## References

[1] National Diabetes Statistics Report | Diabetes | CDC, 2024, https://www.cdc.gov/diabetes/php/data‐research/index.html.

[2] D. M. Williams , H. Jones , and J. W. Stephens , “Personalized Type 2 Diabetes Management: An Update on Recent Advances and Recommendations,” Diabetes, Metabolic Syndrome and Obesity: Targets and Therapy 15 (2022): 281–295, 10.2147/DMSO.S331654. 35153495 PMC8824792

[3] American Diabetes Association Professional Practice Committee , “Pharmacologic Approaches to Glycemic Treatment: Standards of Care in Diabetes‐2025,” Diabetes Care 48, no. 1 Suppl 1 (2025): S181–S206, 10.2337/dc25-S009. 39651989 PMC11635045

[4] H. Sun , P. Saeedi , S. Karuranga , et al., “IDF Diabetes Atlas: Global, Regional and Country‐Level Diabetes Prevalence Estimates for 2021 and Projections for 2045,” Diabetes Research and Clinical Practice 183, no. 2 (2022): 109119, 10.1016/j.diabres.2021.109119. 34879977 PMC11057359

[5] D. Abrahami , E. D'Andrea , H. Yin , et al., “Contemporary Trends in the Utilization of Second‐Line Pharmacological Therapies for Type 2 Diabetes in the United States and the United Kingdom,” Diabetes, Obesity & Metabolism 25, no. 10 (2023): 2980–2988, 10.1111/DOM.15196. PMC10527897 37395339

[6] L. A. Eberly , L. Yang , U. R. Essien , et al., “Racial, Ethnic, and Socioeconomic Inequities in Glucagon‐Like Peptide‐1 Receptor Agonist Use Among Patients With Diabetes in the US,” JAMA Health Forum 2, no. 12 (2021): E214182, 10.1001/JAMAHEALTHFORUM.2021.4182. 35977298 PMC8796881

[7] Z. Majd , H. Chen , M. L. Johnson , K. K. Birtcher , O. Serna , and S. Abughosh , “Real‐World Treatment Patterns in Drug naïve Type 2 Diabetes Population: Initial Combination Therapy vs. Sequential Step‐Therapy,” Journal of the American Pharmacists Association 65, no. 1 (2025): 102295, 10.1016/J.JAPH.2024.102295. 39536802

[8] L. A. Rodriguez , H. Finertie , R. S. Neugebauer , et al., “Race and Ethnicity and Pharmacy Dispensing of SGLT2 Inhibitors and GLP‐1 Receptor Agonists in Type 2 Diabetes,” Lancet Regional Health ‐ Americas 34 (2024): 100759, 10.1016/J.LANA.2024.100759. 38745886 PMC11091531

[9] S. E. Lessing and L. L. Hayman , “Diabetes Care and Management Using Electronic Medical Records: A Systematic Review,” Journal of Diabetes Science and Technology 13, no. 4 (2019): 774–782, 10.1177/1932296818815507. 30556418 PMC6610600

[10] K. Ng , U. Kartoun , H. Stavropoulos , J. A. Zambrano , and P. C. Tang , “Personalized Treatment Options for Chronic Diseases Using Precision Cohort Analytics,” Scientific Reports 11, no. 1 (2021): 1139, 10.1038/S41598-021-80967-5. 33441956 PMC7806725

[11] D. Do , T. Lee , S. K. Peasah , C. B. Good , A. Inneh , and U. Patel , “GLP‐1 Receptor Agonist Discontinuation Among Patients With Obesity and/or Type 2 Diabetes,” JAMA Network Open 7, no. 5 (2024): e2413172, 10.1001/JAMANETWORKOPEN.2024.13172. 38787563 PMC11127113

[12] D. N. Koye , O. Montvida , and S. K. Paul , “Third‐Line Antidiabetic Therapy Intensification Patterns and Glycaemic Control in Patients With Type 2 Diabetes in the USA: A Real‐World Study,” Drugs 80, no. 5 (2020): 477–487, 10.1007/S40265-020-01279-Y. 32141024

[13] O. Montvida , J. Shaw , J. J. Atherton , F. Stringer , and S. K. Paul , “Long‐Term Trends in Antidiabetes Drug Usage in the U.S.: Real‐World Evidence in Patients Newly Diagnosed With Type 2 Diabetes,” Diabetes Care 41, no. 1 (2018): 69–78, 10.2337/DC17-1414. 29109299

[14] S. Mistry , R. Gouripeddi , V. Raman , and J. C. Facelli , “Sequential Data Mining of Infection Patterns as Predictors for Onset of Type 1 Diabetes in Genetically At‐Risk Individuals,” Journal of Biomedical Informatics 142 (2023): 104385, 10.1016/J.JBI.2023.104385. 37169058 PMC10247497

[15] E. I. Benchimol , L. Smeeth , A. Guttmann , et al., “The REporting of Studies Conducted Using Observational Routinely‐Collected Health Data (RECORD) Statement,” PLoS Medicine 12, no. 10 (2015): e1001885, 10.1371/JOURNAL.PMED.1001885. 26440803 PMC4595218

[16] M. B. Palchuk , J. W. London , D. Perez‐Rey , et al., “A Global Federated Real‐World Data and Analytics Platform for Research,” JAMIA Open 6, no. 2 (2023): ooad035, 10.1093/JAMIAOPEN/OOAD035. 37193038 PMC10182857

[17] R. J. Ludwig , M. Anson , H. Zirpel , et al., “A Comprehensive Review of Methodologies and Application to Use the Real‐World Data and Analytics Platform TriNetX,” Frontiers in Pharmacology 16 (2025): 1516126, 10.3389/FPHAR.2025.1516126. 40129946 PMC11931024

[18] A. D. Wiese , C. L. Roumie , J. B. Buse , et al., “Performance of a Computable Phenotype for Identification of Patients With Diabetes Within PCORnet: The Patient‐Centered Clinical Research Network,” Pharmacoepidemiology and Drug Safety 28, no. 5 (2019): 632–639, 10.1002/PDS.4718. 30680840 PMC6615719

[19] G. A. Nichols , J. Desai , J. E. Lafata , et al., “Construction of a Multisite DataLink Using Electronic Health Records for the Identification, Surveillance, Prevention, and Management of Diabetes Mellitus: The SUPREME‐DM Project,” Preventing Chronic Disease 9, no. 6 (2012): 110311, 10.5888/PCD9.110311. PMC3457753 22677160

[20] W. Oh , P. Jayaraman , A. S. Sawant , et al., “Using Sequence Clustering to Identify Clinically Relevant Subphenotypes in Patients With COVID‐19 Admitted to the Intensive Care Unit,” Journal of the American Medical Informatics Association 29, no. 3 (2022): 489–499, 10.1093/JAMIA/OCAB252. 35092685 PMC8800515

[21] A. Großwendt , H. Röglin , and M. Schmidt , “Analysis of Ward's Method,” in Proceedings of the 2019 Annual ACM‐SIAM Symposium on Discrete Algorithms (SODA) (Society for Industrial and Applied Mathematics (SIAM), 2019), 2939–2957, 10.1137/1.9781611975482.182.

[22] C. A. Sugar , R. Sturm , T. T. Lee , et al., “Empirically Defined Health States for Depression From the SF‐12,” Health Services Research 33, no. 4 Pt 1 (1998): 911–928. 9776942 PMC1070293

[23] R. Tibshirani , G. Walther , and T. Hastie , “Estimating the Number of Clusters in a Data Set via the Gap Statistic,” Journal of the Royal Statistical Society, Series B: Statistical Methodology 63, no. 2 (2001): 411–423, 10.1111/1467-9868.00293.

[24] P. V. Kukhareva , R. Gouripeddi , N. Peek , and K. Kawamoto , “Opportunities and Challenges in Using National EHR Networks for AI in Learning Health Systems,” Learning Health Systems 10, no. Suppl 1 (2026): e70090, 10.1002/lrh2.70090. 42253466 PMC13240372

[25] A. Sarpatwari , M. J. Soto , I. Ganguli , C. E. Sloan , F. Goss , and A. D. Sinaiko , “Glucagon‐Like Peptide‐1 Receptor Agonist Order Fills and Out‐Of‐Pocket Costs by Race, Ethnicity, and Indication,” JAMA Health Forum 6, no. 10 (2025): e254258, 10.1001/JAMAHEALTHFORUM.2025.4258. 41071563 PMC12514621

[26] M. Jacobs , Q. Fang , and C. Ellis , “Trends in GLP‐1 Receptor Agonist and SGLT2‐Inhibitor Utilization and Expenditure Between 2017‐2023: Demographic, Income, and Insurance Associations,” Journal of General Internal Medicine, (2026), 10.1007/S11606-026-10408-4. PMC13421679 41984414

[27] A. A. Nargesi , C. Clark , A. Aminorroaya , et al., “Persistence on Novel Cardioprotective Antihyperglycemic Therapies in the United States,” American Journal of Cardiology 196 (2023): 89–98, 10.1016/j.amjcard.2023.03.002. 37012183 PMC11007258

[28] H. Shin , S. Schneeweiss , R. J. Glynn , and E. Patorno , “Trends in First‐Line Glucose‐Lowering Drug Use in Adults With Type 2 Diabetes in Light of Emerging Evidence for SGLT‐2i and GLP‐1RA,” Diabetes Care 44, no. 8 (2021): 1774–1782, 10.2337/DC20-2926. 34385345 PMC8385465

[29] J. Wu , A. Perez , and P. W. Sullivan , “Patterns and Costs Associated With Glucagon‐Like Peptide‐1 Receptor Agonist Use in US Adults With Type 2 Diabetes,” Journal of Managed Care & Specialty Pharmacy 31, no. 10 (2025): 1029–1038, 10.18553/JMCP.2025.31.10.1029. 41004212 PMC12467763

[30] P. Cardoso , J. M. Dennis , and E. R. Pearson , “GLP‐1RA Precision Medicine in People With Type 2 Diabetes: Current Insights and Future Prospects,” Journal of Clinical Investigation 136, no. 2 (2026): e194742, 10.1172/JCI194742. 41542765 PMC12807466

[31] J. M. Dennis , K. G. Young , P. Cardoso , et al., “A Five‐Drug Class Model Using Routinely Available Clinical Features to Optimise Prescribing in Type 2 Diabetes: A Prediction Model Development and Validation Study,” Lancet 405, no. 10480 (2025): 701–714, 10.1016/S0140-6736(24)02617-5. 40020703

[32] J. A. Lamprea‐Montealegre , E. Madden , S. L. Tummalapalli , et al., “Association of Race and Ethnicity With Prescription of SGLT2 Inhibitors and GLP1 Receptor Agonists Among Patients With Type 2 Diabetes in the Veterans Health Administration System,” Journal of the American Medical Association 328, no. 9 (2022): 861–871, 10.1001/JAMA.2022.13885. 36066519 PMC9449794

[33] A. Elhussein , A. Anderson , M. P. Bancks , et al., “Racial/Ethnic and Socioeconomic Disparities in the Use of Newer Diabetes Medications in the Look AHEAD Study,” Lancet Regional Health – Americas 6 (2022): 100111, 10.1016/j.lana.2021.100111. 35291207 PMC8920048

[34] F. Müller , H. Holman , N. Bhangu , J. Kottutt , H. Azhary , and O. Alshaarawy , “Use of Antihyperglycemic Medications Among US People With Limited English Proficiency,” Journal of General Internal Medicine 40, no. 8 (2025): 1803–1810, 10.1007/S11606-025-09385-X. 39875767 PMC12119434

[35] P. V. Kukhareva , M. J. O'Brien , D. C. Malone , et al., “Documentation of Social Determinants of Health for Patients With Type 2 Diabetes in Epic Cosmos,” JAMIA Open 8, no. 5 (2025): ooaf095, 10.1093/JAMIAOPEN/OOAF095. 40918938 PMC12410986

[36] S. J. Pilla , J. B. Segal , G. C. Alexander , C. M. Boyd , and N. M. Maruthur , “Differences in National Diabetes Treatment Patterns and Trends Between Older and Younger Adults,” Journal of the American Geriatrics Society 67, no. 5 (2019): 1066–1073, 10.1111/jgs.15790. 30703251 PMC6488408

[37] K. Vafeidou , O. Psoma , E. Apostolidis , et al., “Clinical Inertia in SGLT2 Inhibitor Use Among Elderly Patients With Type 2 Diabetes and Chronic Kidney Disease: A Comparison of Regional and University Hospital Practice,” Geriatrics 10, no. 6 (2025): 144, 10.3390/GERIATRICS10060144. 41283455 PMC12641619

[38] M. H. Jensen , M. Kjolby , O. Hejlesen , P. E. Jakobsen , and P. Vestergaard , “Risk of Major Adverse Cardiovascular Events, Severe Hypoglycemia, and All‐Cause Mortality for Widely Used Antihyperglycemic Dual and Triple Therapies for Type 2 Diabetes Management: A Cohort Study of All Danish Users,” Diabetes Care 43, no. 6 (2020): 1209–1218, 10.2337/dc19-2535. 32238426

[39] A. D. Misra‐Hebert , K. M. Pantalone , X. Ji , et al., “Patient Characteristics Associated With Severe Hypoglycemia in a Type 2 Diabetes Cohort in a Large, Integrated Health Care System From 2006 to 2015,” Diabetes Care 41, no. 6 (2018): 1164–1171, 10.2337/dc17-1834. 29549082

