# Supplementary material for: Prescribing Trajectories in Type 2 Diabetes in the United States, 2019–2024
Source: Diabetes Obes Metab. 2026 Jun 17;28(9):8172–88. doi: 10.1111/dom.71013 (PMC13448853; doi:10.1111/dom.71013)
Supplement: Supplementary file 1 [file DOM-28-8172-s001.docx]

**Supplement 1. Figures and Tables**

**Prescribing Trajectories in Type 2 Diabetes in the United States, 2019-2024**

Tobias S. Lux^1^, Dongkun Lee^1^, Jeff M. Phillips, PhD^1^, Julio C. Facelli, PhD^2,3^, Daniel C Malone, PhD^4^, Matt Wahl^5^, MD, Deepika Reddy, MD^5^, Farrant Sakaguchi, MD, MS^6^, Ramkiran Gouripeddi, MBBS, MS^2,3^, Alexander S. Millar, MS^2^, Matthew J. O'Brien, MD, MSc^7^, Joshua Choi, MD^2^, Jittrapol Sutejitsiri^2^, Kensaku Kawamoto, MD, PhD, MHS^2^, Polina V. Kukhareva, PhD, MPH^2^

^1^ - Kahler School of Computing, University of Utah, Salt Lake City, UT 84108, United States,

^2^ - Department of Biomedical Informatics, University of Utah, Salt Lake City, UT 84108, United States,

^3^ - Utah Clinical and Translational Institute, University of Utah, Salt Lake City, UT 84108, United States,

^4^ - Department of Pharmacotherapy, University of Utah, Salt Lake City, UT 84108, United States,

^5^ - Diabetes and Endocrinology Center, University of Utah, Salt Lake City, UT 84108, United States,

^6^ - Department of Family and Preventative Medicine, University of Utah, Salt Lake City, UT 84108, United States,

^7^ - Department of General Internal Medicine, Northwestern University, Chicago, IL 60611, United States

**eTable 1. Clinical Codes Used in the Study**

| Analytic Use of Code(s) | Code System | Code(s) |
| --- | --- | --- |
| BMI | LOINC | 39156-5 |
|  |  | 41909-3 |
|  |  | LP35925-4 |
| HbA1c | LOINC | 4548-4 |
|  |  | 17856-6 |
|  |  | 4549-2 |
| T2D Diagnosis | ICD-10-CM | E11* |
| Diabetes mellitus in pregnancy, childbirth, and the puerperium | ICD-10-CM | O24* |
| Heart Failure | ICD-10-CM | I50* |
|  |  | I11.0 |
|  |  | I13.0 |
|  |  | I13.2 |
| Chronic Kidney Disease | ICD-10-CM | N18.1 |
|  |  | N18.2 |
|  |  | N18.30 |
|  |  | N18.31 |
|  |  | N18.4 |
|  |  | N18.5 |
|  |  | N18.6 |
|  |  | N18.9 |
|  |  | I12* |
|  |  | E11.22 |
| Glucose-lowering Medications | ATC | A10* |

**Note:** Codes shown were used to define clinical conditions, laboratory measurements, and medication classes for cohort construction and analysis. (*) indicate inclusion of all subordinate codes within the specified classification.

**eTable 2. Primary Analytic Cohort vs. Early Disengagement Clusters**

| Characteristic | Primary Cohort (n=9,327) | Disengagement (n=9,325) | SMD |
| --- | --- | --- | --- |
| N | 9,327 | 9,325 |  |
| Demographics |  |  |  |
| Female sex | n=4,484, 48.1% | n=4,564, 48.9% | 0.017 |
| Age category, years |  |  |  |
| - 18-45 | n=1,567, 16.8% | n=1,165, 12.5% | 0.122 |
| - 45-54 | n=2,327, 24.9% | n=1,756, 18.8% | 0.148 |
| - 55-64 | n=2,923, 31.3% | n=2,688, 28.8% | 0.055 |
| - 65+ | n=2,510, 26.9% | n=3,716, 39.8% | 0.277 |
| Race/ethnicity |  |  |  |
| - White, NH | n=5,686, 61.0% | n=5,571, 59.7% | 0.025 |
| - Hispanic/Latinx | n=863, 9.3% | n=892, 9.6% | 0.011 |
| - Asian, NH | n=605, 6.5% | n=509, 5.5% | 0.043 |
| - African American/Black, NH | n=1,657, 17.8% | n=1,875, 20.1% | 0.060 |
| - Other, NH | n=516, 5.5% | n=478, 5.1% | 0.018 |
| US Region |  |  |  |
| - Midwest | n=2,000, 21.4% | n=1,549, 16.6% | 0.123 |
| - South | n=3,269, 35.0% | n=3,653, 39.2% | 0.085 |
| - West | n=1,255, 13.5% | n=1,069, 11.5% | 0.060 |
| - Northeast | n=2,803, 30.1% | n=3,054, 32.8% | 0.058 |
| Clinical Characteristics |  |  |  |
| Baseline HbA1c (pre-treatment, 2019) |  |  |  |
| Mean ± SD (%) | 8.3 ± 2.2 | 7.7 ± 2.2 | 0.280 |
| - <6.4 | n=502, 5.4% | n=964, 10.3% | 0.185 |
| - 6.5-7.9 | n=2,701, 29.0% | n=1,719, 18.4% | 0.249 |
| - 8.0-8.9 | n=656, 7.0% | n=376, 4.0% | 0.132 |
| - >= 9.0 | n=1,658, 17.8% | n=840, 9.0% | 0.260 |
| - Missing | n=3,810, 40.8% | n=5,426, 58.2% | 0.352 |
| Baseline BMI (pre-treatment, 2019, kg/m²) |  |  |  |
| Mean ± SD (kg/m²) | 34.5 ± 6.6 | 32.6 ± 6.8 | 0.281 |
| - <24.9 | n=262, 2.8% | n=501, 5.4% | 0.130 |
| - 25.0-29.9 | n=885, 9.5% | n=1,126, 12.1% | 0.083 |
| - 30.0-34.9 | n=1,335, 14.3% | n=1,156, 12.4% | 0.056 |
| - 35.0-39.9 | n=1,030, 11.0% | n=819, 8.8% | 0.076 |
| - >= 40 | n=990, 10.6% | n=668, 7.2% | 0.121 |
| - Missing | n=4,825, 51.7% | n=5,055, 54.2% | 0.050 |
| Comorbidities before June 1, 2019 |  |  |  |
| - Heart Failure | n=574, 6.2% | n=965, 10.3% | 0.153 |
| - Chronic Kidney Disease | n=710, 7.6% | n=1,053, 11.3% | 0.126 |

**Note:** Values expressed as N (%) or mean ± SD. Standardized mean differences (SMD) quantify the magnitude of difference between groups independent of sample size; Baseline HbA1c and BMI represent the closest recorded value at or before each patient's first glucose-lowering medication prescription within 2019. 'Missing' indicates patients without a qualifying pre-treatment value in that window.

| Therapy Category | Clusters (n) | Patients (n, %) | Clinical Interpretation | Dominant Drug Classes | BMI / HbA1c Trends |
| --- | --- | --- | --- | --- | --- |
| Monotherapy | 5 | 2,759 (29.6%) | Single-agent therapy maintained throughout follow-up; clusters differ primarily by follow-up intensity | Metformin (predominant, 2,533 patients); insulin (226 patients) | BMI ↓ −1.38 kg/m² (95% CI, −1.60 to −1.16) and HbA1c ↓ −0.16 pp (−0.27 to −0.06) across metformin clusters; insulin cluster has higher HF/CKD comorbidity burden |
| Dual Therapy | 8 | 1,951 (20.9%) | Stable two-drug regimens maintained throughout follow-up | Metformin in combination with sulfonylurea, SGLT2i, DPP-4i, TZD, or insulin | BMI ↓ ranging −0.89 kg/m² (MET+SUL) to −2.12 kg/m² (MET+SGLT2i); modest but statistically significant HbA1c reductions across most clusters |
| Complex Therapy | 3 | 527 (5.7%) | Three- or more drug-class regimens, including insulin-intensified triple therapies | MET+SGLT2i+GLP-1 RA; MET+SUL+INS; INS+DPP-4i+MET | MET+SGLT2i+GLP-1 RA: BMI ↓ −1.63 kg/m² (−2.28 to −0.98), HbA1c NS. Insulin-based triple therapies: minimal/NS changes |
| Variant Therapy | 3 | 1,225 (13.0%) | Atypical patterns not aligning with standard categories — alternating, transitional, or low-frequency add-on use | Insulin↔metformin alternation; sulfonylurea-based; DPP-4i-based | Largest cluster (INS↔MET): BMI ↓ −1.68 kg/m² (−2.23 to −1.12), HbA1c ↓ −0.80 pp (−1.10 to −0.50). Other clusters: modest BMI ↓, HbA1c NS |
| GLP-1 RA Therapy | 11 | 2,865 (30.7%) | Trajectories featuring GLP-1 RA or GIP/GLP-1 RA prescribing, typically as a transition from initial therapy (most commonly metformin) | GLP-1 RA and GIP/GLP-1 RA, alone or in combination with metformin, insulin, or SGLT2i | Substantial BMI ↓ (MET→GLP-1 RA: −2.20 kg/m² [−2.55 to −1.85]; MET→GIP/GLP-1 RA: −3.43 kg/m² [−4.08 to −2.78]) and meaningful HbA1c ↓ (−0.44 to −0.67 pp); patients generally had higher baseline BMI |
| Early Disengagement (analyzed separately) | 10 | 9,325 | Medication data concentrated in first 1–2 study intervals with minimal subsequent activity; consistent with early care disengagement or transition out of TriNetX | Heterogeneous; sparse prescribing across all classes | Not assessed (insufficient longitudinal data for trajectory modeling) |

**eTable 3. Practical Summary of Higher-Level Prescribing Trajectory Categories**

**Note:** Percentages are relative to the primary analytic cohort (n = 9,327); the Early Disengagement group was analyzed separately (eFigure 2) and its count reported in absolute terms. BMI and HbA1c changes are adjusted 2019–2024 estimates and represent descriptive associations rather than treatment effects. **Abbreviations:** CI, confidence interval; DPP-4i, dipeptidyl peptidase-4 inhibitor; GIP/GLP-1 RA, dual glucose-dependent insulinotropic polypeptide and glucagon-like peptide-1 RA; GLP-1 RA, glucagon-like peptide-1 RA; INS, insulin; MET, metformin; NS, not statistically significant; pp, percentage points; SGLT2i, sodium-glucose cotransporter-2 inhibitor; SUL, sulfonylurea; TZD, thiazolidinedione.

**eTable 4. Most Prevalent Prescriptions and Comorbidities in Each Cluster**

| Cluster | # | Top Rx | Second Rx | HF | CKD |
| --- | --- | --- | --- | --- | --- |
| Mono: MET (Low F/U) | 1 | MET (99.2) | Insulin, MET (0.3) | 6.9 | 12.8 |
| Mono: MET (Low F/U) | 2 | MET (93.4) | GLP, 1 (1.6) | 6.6 | 14.1 |
| Mono: MET (High F/U) | 3 | MET (98.4) | Insulin, MET (1.1) | 10.6 | 15.1 |
| Mono: MET (Very Low F/U) | 4 | MET (91.2) | Insulin (1.9) | 6.9 | 10.7 |
| Mono: INS | 5 | Insulin (92.9) | GLP, 1, Insulin (2.7) | 46 | 58.4 |
| Dual: MET+SUL | 1 | MET (48.6) | MET, SUL (24.8) | 12.8 | 18.3 |
| Dual: MET+SUL | 2 | MET, SUL (42.7) | MET, SGLT2, SUL (9.9) | 11.6 | 22.5 |
| Dual: MET+DPP-4i | 3 | DPP, 4, MET (44.2) | MET (22.3) | 9.9 | 19.1 |
| Dual: MET+SGLT2i | 4 | MET, SGLT2 (53.5) | MET (32.7) | 12.8 | 14.2 |
| Dual: MET+INS | 5 | Insulin, MET (60.4) | Insulin (16.0) | 24.1 | 34.2 |
| Dual: INS+SGLT2i (Low F/U) | 6 | Insulin (39.5) | Insulin, SGLT2 (16.2) | 46.5 | 48.1 |
| Dual: MET+TZD | 7 | MET (22.2) | MET, TZD (20.6) | 8.7 | 20.6 |
| Dual: MET+SUL (Declining F/U) | 8 | MET, SUL (49.6) | MET (23.1) | 13.7 | 18.8 |
| Complex: MET+SGLT2i+GLP-1 | 1 | MET (48.4) | MET, SGLT2 (13.5) | 22.1 | 21.2 |
| Complex: MET+SUL+INS | 2 | Insulin (13.1) | Insulin, MET, SUL (13.1) | 25.5 | 39.2 |
| Complex: INS+DPP-4i+MET | 3 | DPP, 4, Insulin, MET (27.4) | DPP, 4, Insulin (25.8) | 22.6 | 45.2 |
| Variant: INS↔MET | 1 | Insulin (34.4) | Insulin, MET (16.9) | 31.3 | 29.8 |
| Variant: SUL-based | 2 | SUL (44.1) | GLP, 1 (10.3) | 17.4 | 31 |
| Variant: DPP-4i-based (Low F/U) | 3 | DPP, 4 (40.2) | DPP, 4, MET (12.8) | 16.8 | 34 |
| GLP-1: MET→GLP-1 RA (Early) | 1 | MET (39.8) | GLP, 1, MET (33.9) | 9.7 | 13.9 |
| GLP-1: MET+INS→GLP-1 RA | 2 | GLP, 1 (67.1) | MET (10.4) | 10.8 | 18.6 |
| GLP-1: MET→GLP-1 RA (Late) | 3 | MET (76.3) | GLP, 1, MET (13.8) | 6.6 | 11.1 |
| GLP-1: MET→GIP/GLP-1 RA | 4 | MET (44.3) | GIP/GLP, 1 (23.2) | 8.3 | 9.7 |
| GLP-1: MET+INS→INS+GLP-1 | 5 | GLP, 1, Insulin (18.5) | Insulin (17.3) | 19.6 | 30.6 |
| GLP-1: Early GLP-1 RA | 6 | GLP, 1 (91.3) | MET (2.1) | 6.6 | 16.9 |
| GLP-1: SGLT2→SGLT2+GLP-1 | 7 | SGLT2 (35.8) | GLP, 1, SGLT2 (19.8) | 19.8 | 18.2 |
| GLP-1: INS+GLP-1 RA | 8 | GLP, 1, Insulin (49.4) | Insulin (15.7) | 23.5 | 33.1 |
| GLP-1: MET+INS→+GLP-1 RA | 9 | GLP, 1, Insulin, MET (33.6) | GLP, 1, Insulin, MET, SGLT2 (16.1) | 19.6 | 21.7 |
| GLP-1: MET+SGLT2i+GLP-1 RA | 10 | GLP, 1, MET, SGLT2 (38.3) | GLP, 1, MET (12.8) | 11.7 | 25.5 |
| GLP-1: MET+SUL→GLP-1 RA | 11 | GLP, 1, MET, SUL (26.4) | MET, SUL (18.4) | 10.3 | 19.5 |

Note: Values expressed as %. Heart failure (HF) and chronic kidney disease (CKD) were determined using International Classification of Diseases, Tenth Revision, Clinical Modification (ICD-10-CM) codes and reflect records from throughout the entire study period. Glucagon-like peptide-1 (GLP-1) therapy is defined as a treatment pattern that includes a GLP-1 receptor agonist (RA) component. Variant therapy denotes treatment patterns that do not align with the other predefined group profiles. Abbreviations: CI, confidence interval; DPP-4i, dipeptidyl peptidase-4 inhibitor; F/U, follow-up; GIP/GLP-1 RA, dual glucose-dependent insulinotropic polypeptide and glucagon-like peptide-1 RA; GLP-1 RA, glucagon-like peptide-1 RA; INS, insulin; MET, metformin; NH, non-Hispanic; OR, odds ratio; SGLT2i, sodium-glucose cotransporter-2 inhibitor; SUL, sulfonylurea; TZD, thiazolidinedione.

**eTable 5. Adjusted Odds Ratios for HF/CKD by Cluster**

| Cluster | # | n | OR (95% CI) | p-value (BH) |
| --- | --- | --- | --- | --- |
| Mono: MET (Low F/U) | 1 | 765 | 0.54 (0.35–0.79) | 0.013* |
| Mono: MET (Low F/U) | 2 | 707 | 0.49 (0.30–0.75) | 0.009** |
| Mono: MET (High F/U) | 3 | 695 | 0.87 (0.59–1.23) | 0.651 |
| Mono: MET (Very Low F/U) | 4 | 363 | 0.51 (0.25–0.91) | 0.103 |
| Mono: INS | 5 | 226 | 2.29 (1.43–3.50) | 0.002** |
| Dual: MET+SUL | 1 | 525 | 0.76 (0.46–1.18) | 0.424 |
| Dual: MET+SUL | 2 | 302 | 0.58 (0.26–1.10) | 0.268 |
| Dual: MET+DPP-4i | 3 | 283 | 0.68 (0.32–1.25) | 0.437 |
| Dual: MET+SGLT2i | 4 | 226 | 1.51 (0.83–2.54) | 0.291 |
| Dual: MET+INS | 5 | 187 | 0.78 (0.33–1.56) | 0.725 |
| Dual: INS+SGLT2i (Low F/U) | 6 | 185 | 2.07 (1.19–3.36) | 0.023* |
| Dual: MET+TZD | 7 | 126 | 0.75 (0.26–1.67) | 0.725 |
| Dual: MET+SUL (Declining F/U) | 8 | 117 | 0.34 (0.06–1.08) | 0.270 |
| Complex: MET+SGLT2i+GLP-1 | 1 | 312 | 1.79 (1.14–2.69) | 0.027* |
| Complex: MET+SUL+INS | 2 | 153 | 1.11 (0.46–2.22) | 0.890 |
| Complex: INS+DPP-4i+MET | 3 | 62 | 2.37 (0.90–5.18) | 0.126 |
| Variant: INS↔MET | 1 | 514 | 1.45 (0.98–2.07) | 0.128 |
| Variant: SUL-based | 2 | 390 | 1.60 (1.05–2.35) | 0.068 |
| Variant: DPP-4i-based (Low F/U) | 3 | 321 | 2.44 (1.64–3.52) | <0.001*** |
| GLP-1: MET→GLP-1 RA (Early) | 1 | 628 | 1.08 (0.72–1.57) | 0.836 |
| GLP-1: MET+INS→GLP-1 RA | 2 | 424 | 0.96 (0.58–1.51) | 0.933 |
| GLP-1: MET→GLP-1 RA (Late) | 3 | 333 | 0.63 (0.31–1.13) | 0.300 |
| GLP-1: MET→GIP/GLP-1 RA | 4 | 289 | 0.84 (0.41–1.51) | 0.768 |
| GLP-1: MET+INS→INS+GLP-1 | 5 | 271 | 1.16 (0.62–1.97) | 0.779 |
| GLP-1: Early GLP-1 RA | 6 | 241 | 0.96 (0.47–1.74) | 0.935 |
| GLP-1: SGLT2→SGLT2+GLP-1 | 7 | 187 | 1.21 (0.59–2.21) | 0.750 |
| GLP-1: INS+GLP-1 RA | 8 | 166 | 1.24 (0.55–2.40) | 0.750 |
| GLP-1: MET+INS→+GLP-1 RA | 9 | 143 | 0.31 (0.05–0.97) | 0.215 |
| GLP-1: MET+SGLT2i+GLP-1 RA | 10 | 94 | 1.55 (0.54–3.50) | 0.543 |
| GLP-1: MET+SUL→GLP-1 RA | 11 | 87 | 1.17 (0.35–2.84) | 0.874 |

**Note:** Odds ratios derived from the multivariable one-vs-rest logistic regression models also reported in Table 2 of the main manuscript. Models adjusted for age category, sex, race and ethnicity, BMI category, HbA1c category, and a composite HF/CKD indicator. P-values are Benjamini–Hochberg (BH) adjusted; significance markers: *p<0.05, **p<0.01, ***p<0.001.

**eTable 6. Patient counts used for adjusted BMI and HbA1c trajectories**

| Cluster | # | Total Patients | BMI Recorded Each Year | HbA1c Recorded Each Year |
| --- | --- | --- | --- | --- |
| Mono: MET (Low F/U) | 1 | 766 | 295 (39%) | 402 (52%) |
| Mono: MET (Low F/U) | 2 | 708 | 286 (40%) | 335 (47%) |
| Mono: MET (High F/U) | 3 | 696 | 303 (44%) | 351 (50%) |
| Mono: MET (Very Low F/U) | 4 | 363 | 106 (29%) | 118 (33%) |
| Mono: INS | 5 | 226 | 94 (42%) | 79 (35%) |
| Dual: MET+SUL | 1 | 525 | 210 (40%) | 239 (46%) |
| Dual: MET+SUL | 2 | 302 | 124 (41%) | 159 (53%) |
| Dual: MET+DPP-4i | 3 | 283 | 99 (35%) | 141 (50%) |
| Dual: MET+SGLT2i | 4 | 226 | 92 (41%) | 133 (59%) |
| Dual: MET+INS | 5 | 187 | 73 (39%) | 78 (42%) |
| Dual: INS+SGLT2i (Low F/U) | 6 | 185 | 55 (30%) | 56 (30%) |
| Dual: MET+TZD | 7 | 126 | 38 (30%) | 71 (56%) |
| Dual: MET+SUL (Declining F/U) | 8 | 117 | 36 (31%) | 46 (39%) |
| Complex: MET+SGLT2i+GLP-1 RA | 1 | 312 | 111 (36%) | 130 (42%) |
| Complex: MET+SUL+INS | 2 | 153 | 72 (47%) | 79 (52%) |
| Complex: INS+DPP-4i+MET | 3 | 62 | 26 (42%) | 34 (55%) |
| Variant: INS↔MET | 1 | 514 | 153 (30%) | 158 (31%) |
| Variant: SUL-based | 2 | 390 | 169 (43%) | 188 (48%) |
| Variant: DPP-4i-based (Low F/U) | 3 | 321 | 101 (31%) | 129 (40%) |
| GLP-1: MET→GLP-1 RA (Early) | 1 | 628 | 245 (39%) | 325 (52%) |
| GLP-1: MET+INS→GLP-1 RA | 2 | 425 | 128 (30%) | 110 (26%) |
| GLP-1: MET→GLP-1 RA (Late) | 3 | 333 | 152 (46%) | 178 (53%) |
| GLP-1: MET→GIP/GLP-1 RA | 4 | 289 | 112 (39%) | 148 (51%) |
| GLP-1: MET+INS→INS+GLP-1 RA | 5 | 271 | 99 (37%) | 113 (42%) |
| GLP-1: Early GLP-1 RA | 6 | 242 | 107 (44%) | 126 (52%) |
| GLP-1: SGLT2→SGLT2+GLP-1 RA | 7 | 187 | 76 (41%) | 84 (45%) |
| GLP-1: INS+GLP-1 RA | 8 | 166 | 89 (54%) | 90 (54%) |
| GLP-1: MET+INS→+GLP-1 RA | 9 | 143 | 70 (49%) | 79 (55%) |
| GLP-1: MET+SGLT2i+GLP-1 RA | 10 | 94 | 36 (38%) | 59 (63%) |
| GLP-1: MET+SUL→GLP-1 RA | 11 | 87 | 38 (44%) | 43 (49%) |

**Note: Values expressed as N (%).** This table reports, by cluster, the number and proportion of patients eligible for inclusion in age-adjusted BMI and HbA1c trajectory analyses. Eligibility required at least one recorded BMI or HbA1c measurement in *each* calendar year of the study period (2019–2024). Percentages are calculated relative to the total number of patients in each cluster. BMI and HbA1c eligibility were assessed independently; patients were not required to have complete data for both measures. **Abbreviations:** **CI**, confidence interval; **DPP-4i**, dipeptidyl peptidase-4 inhibitor; **F/U**, follow-up; **GIP/GLP-1 RA**, dual glucose-dependent insulinotropic polypeptide and glucagon-like peptide-1 RA; **GLP-1 RA**, glucagon-like peptide-1 RA; **INS**, insulin; **MET**, metformin; **NH**, non-Hispanic; **OR**, odds ratio; **SGLT2i**, sodium-glucose cotransporter-2 inhibitor; **SUL**, sulfonylurea; **TZD**, thiazolidinedione.

**eTable 7. Patient Characteristics by Race/Ethnicity**

| Characteristic | White, NH (N=5,686) | Black, NH (N=1,657) | Hispanic/Latinx (N=863) | Asian, NH (N=605) | Other, NH (N=516) |
| --- | --- | --- | --- | --- | --- |
| Demographics |  |  |  |  |  |
| - Female sex | 2,510 (44.1%) | 965 (58.2%) | 473 (54.8%) | 288 (47.6%) | 248 (48.1%) |
| Age category, years |  |  |  |  |  |
| - 18-44 | 769 (13.5%) | 367 (22.1%) | 231 (26.8%) | 101 (16.7%) | 99 (19.2%) |
| - 45-54 | 1,303 (22.9%) | 476 (28.7%) | 260 (30.1%) | 146 (24.1%) | 142 (27.5%) |
| - 55-64 | 1,868 (32.9%) | 532 (32.1%) | 210 (24.3%) | 166 (27.4%) | 147 (28.5%) |
| - 65+ | 1,746 (30.7%) | 282 (17.0%) | 162 (18.8%) | 192 (31.7%) | 128 (24.8%) |
| Baseline HbA1c (pre-treatment, 2019) |  |  |  |  |  |
| - <6.4 | 322 (5.7%) | 93 (5.6%) | 35 (4.1%) | 31 (5.1%) | 21 (4.1%) |
| - 6.5-7.9 | 1,685 (29.6%) | 481 (29.0%) | 184 (21.3%) | 218 (36.0%) | 133 (25.8%) |
| - 8.0-8.9 | 435 (7.7%) | 91 (5.5%) | 51 (5.9%) | 46 (7.6%) | 33 (6.4%) |
| - >= 9.0 | 1,064 (18.7%) | 309 (18.6%) | 140 (16.2%) | 71 (11.7%) | 74 (14.3%) |
| - Missing | 2,180 (38.3%) | 683 (41.2%) | 453 (52.5%) | 239 (39.5%) | 255 (49.4%) |
| Baseline BMI (pre-treatment, 2019) |  |  |  |  |  |
| - <24.9 | 107 (1.9%) | 38 (2.3%) | 35 (4.1%) | 61 (10.1%) | 20 (3.9%) |
| - 25.0-29.9 | 462 (8.1%) | 125 (7.5%) | 115 (13.3%) | 102 (16.9%) | 78 (15.1%) |
| - 30.0-34.9 | 817 (14.4%) | 203 (12.3%) | 171 (19.8%) | 80 (13.2%) | 64 (12.4%) |
| - 35.0-39.9 | 662 (11.6%) | 189 (11.4%) | 103 (11.9%) | 25 (4.1%) | 52 (10.1%) |
| - >= 40 | 621 (10.9%) | 195 (11.8%) | 104 (12.1%) | 15 (2.5%) | 58 (11.2%) |
| - Missing | 3,017 (53.1%) | 907 (54.7%) | 335 (38.8%) | 322 (53.2%) | 244 (47.3%) |
| Comorbidities before June 1, 2019 |  |  |  |  |  |
| - Heart Failure | 317 (5.6%) | 117 (7.1%) | 37 (4.3%) | 31 (5.1%) | 19 (3.7%) |
| - Chronic Kidney Disease | 340 (6.0%) | 146 (8.8%) | 53 (6.1%) | 52 (8.6%) | 25 (4.8%) |

**Note:** Values are summarized as N (%). Within each race/ethnicity column, percentages are calculated relative to the column total (denominator shown in each column header). Baseline glycated hemoglobin (HbA1c) and body mass index (BMI) represent the closest recorded value at or before each patient's first glucose-lowering medication (GLM) prescription, restricted to a 183-day pre-treatment window within 2019; patients without a qualifying pre-treatment value are reported as missing. Comorbidities indicate documented heart failure (HF) or chronic kidney disease (CKD) between January 1 and June 1, 2019. Race and ethnicity categories reflect self-reported race as recorded in the electronic health record. Abbreviations: BMI, body mass index; CKD, chronic kidney disease; GLM, glucose-lowering medication; HbA1c, glycated hemoglobin; HF, heart failure; NH, Non-Hispanic.

**eTable 8. HbA1c Trajectory-Eligible vs. Ineligible Patients.**

| Characteristic | HbA1c-Eligible | HbA1c-Ineligible | SMD |
| --- | --- | --- | --- |
| N | 4,281 | 5,046 |  |
| Demographics |  |  |  |
| - Female sex | n=2,054, 48.0% | n=2,430, 48.2% | 0.004 |
| Age (years), mean ± SD | 57.3 ± 11.6 | 55.4 ± 12.3 | 0.158 |
| Age category, years |  |  |  |
| - 18-45 | n=603, 14.1% | n=964, 19.1% | 0.135 |
| - 45-54 | n=1,005, 23.5% | n=1,322, 26.2% | 0.063 |
| - 55-64 | n=1,422, 33.2% | n=1,501, 29.7% | 0.075 |
| - 65+ | n=1,251, 29.2% | n=1,259, 25.0% | 0.096 |
| Race/ethnicity |  |  |  |
| - White, NH | n=2,816, 65.8% | n=2,870, 56.9% | 0.184 |
| - Hispanic/Latinx | n=312, 7.3% | n=551, 10.9% | 0.126 |
| - Asian, NH | n=340, 7.9% | n=265, 5.3% | 0.109 |
| - African American/Black, NH | n=604, 14.1% | n=1,053, 20.9% | 0.179 |
| - Other, NH | n=209, 4.9% | n=307, 6.1% | 0.053 |
| US Region |  |  |  |
| - Midwest | n=1,072, 25.0% | n=928, 18.4% | 0.162 |
| - South | n=1,321, 30.9% | n=1,948, 38.6% | 0.163 |
| - West | n=519, 12.1% | n=736, 14.6% | 0.072 |
| - Northeast | n=1,369, 32.0% | n=1,434, 28.4% | 0.078 |
| Clinical Characteristics |  |  |  |
| Baseline HbA1c (pre-treatment, 2019) |  |  |  |
| Mean ± SD (%) | 8.2 ± 2.1 | 8.4 ± 2.3 | 0.088 |
| - <6.4 | n=277, 6.5% | n=225, 4.5% | 0.089 |
| - 6.5-7.9 | n=1,595, 37.3% | n=1,106, 21.9% | 0.341 |
| - 8.0-8.9 | n=382, 8.9% | n=274, 5.4% | 0.136 |
| - >= 9.0 | n=881, 20.6% | n=777, 15.4% | 0.135 |
| - Missing | n=1,146, 26.8% | n=2,664, 52.8% | 0.552 |
| Baseline BMI (pre-treatment, 2019, kg/m²) |  |  |  |
| Mean ± SD (kg/m²) | 34.3 ± 6.4 | 34.7 ± 6.8 | 0.064 |
| - <24.9 | n=133, 3.1% | n=129, 2.6% | 0.033 |
| - 25.0-29.9 | n=437, 10.2% | n=448, 8.9% | 0.045 |
| - 30.0-34.9 | n=680, 15.9% | n=655, 13.0% | 0.083 |
| - 35.0-39.9 | n=513, 12.0% | n=517, 10.2% | 0.055 |
| - >= 40 | n=459, 10.7% | n=531, 10.5% | 0.006 |
| - Missing | n=2,059, 48.1% | n=2,766, 54.8% | 0.135 |
| Comorbidities before June 1, 2019 |  |  |  |
| - Heart Failure | n=208, 4.9% | n=313, 6.2% | 0.059 |
| - Chronic Kidney Disease | n=293, 6.8% | n=323, 6.4% | 0.018 |

**Note:** Demographic and baseline clinical characteristics of primary-cohort patients eligible vs. ineligible for HbA1c trajectory analysis. Eligibility required at least one recorded HbA1c value in every calendar year from 2019 through 2024. Standardized mean differences (SMDs) quantify the magnitude of difference between groups independent of sample size. Baseline HbA1c and BMI represent the closest recorded value at or before each patient's first GLM prescription within 2019; "Missing" indicates patients without a qualifying pre-treatment value.

**eTable 9. BMI Trajectory-Eligible vs. Ineligible Patients.**

| Characteristic | BMI-Eligible | BMI-Ineligible | SMD |
| --- | --- | --- | --- |
| N | 3,595 | 5,732 |  |
| Demographics |  |  |  |
| - Female sex | n=1,760, 49.0% | n=2,724, 47.5% | 0.029 |
| Age (years), mean ± SD | 57.5 ± 11.8 | 55.5 ± 12.1 | 0.166 |
| Age category, years |  |  |  |
| - 18-45 | n=511, 14.2% | n=1,056, 18.4% | 0.114 |
| - 45-54 | n=832, 23.1% | n=1,495, 26.1% | 0.068 |
| - 55-64 | n=1,155, 32.1% | n=1,768, 30.8% | 0.028 |
| - 65+ | n=1,097, 30.5% | n=1,413, 24.7% | 0.131 |
| Race/ethnicity |  |  |  |
| - White, NH | n=2,238, 62.3% | n=3,448, 60.2% | 0.043 |
| - Hispanic/Latinx | n=376, 10.5% | n=487, 8.5% | 0.067 |
| - Asian, NH | n=218, 6.1% | n=387, 6.8% | 0.028 |
| - African American/Black, NH | n=567, 15.8% | n=1,090, 19.0% | 0.086 |
| - Other, NH | n=196, 5.5% | n=320, 5.6% | 0.006 |
| US Region |  |  |  |
| - Midwest | n=1,343, 37.4% | n=657, 11.5% | 0.632 |
| - South | n=689, 19.2% | n=2,580, 45.0% | 0.576 |
| - West | n=489, 13.6% | n=766, 13.4% | 0.007 |
| - Northeast | n=1,074, 29.9% | n=1,729, 30.2% | 0.006 |
| Clinical Characteristics |  |  |  |
| Baseline HbA1c (pre-treatment, 2019, %) |  |  |  |
| Mean ± SD (%) | 8.2 ± 2.1 | 8.5 ± 2.2 | 0.127 |
| - <6.4 | n=220, 6.1% | n=282, 4.9% | 0.053 |
| - 6.5-7.9 | n=1,240, 34.5% | n=1,461, 25.5% | 0.197 |
| - 8.0-8.9 | n=277, 7.7% | n=379, 6.6% | 0.042 |
| - >= 9.0 | n=632, 17.6% | n=1,026, 17.9% | 0.008 |
| - Missing | n=1,226, 34.1% | n=2,584, 45.1% | 0.226 |
| Baseline BMI (pre-treatment, 2019, kg/m²) |  |  |  |
| Mean ± SD (kg/m²) | 34.4 ± 6.5 | 34.8 ± 6.9 | 0.064 |
| - <24.9 | n=184, 5.1% | n=78, 1.4% | 0.213 |
| - 25.0-29.9 | n=615, 17.1% | n=270, 4.7% | 0.406 |
| - 30.0-34.9 | n=950, 26.4% | n=385, 6.7% | 0.550 |
| - 35.0-39.9 | n=735, 20.4% | n=295, 5.1% | 0.470 |
| - >= 40 | n=664, 18.5% | n=326, 5.7% | 0.400 |
| - Missing | n=447, 12.4% | n=4,378, 76.4% | 1.681 |
| Comorbidities before June 1, 2019 |  |  |  |
| - Heart Failure | n=234, 6.5% | n=287, 5.0% | 0.065 |
| - Chronic Kidney Disease | n=292, 8.1% | n=324, 5.7% | 0.098 |

**Note:** Demographic and baseline clinical characteristics of primary-cohort patients eligible vs. ineligible for BMI trajectory analysis. Eligibility required at least one recorded BMI value in every calendar year from 2019 through 2024. Other notes as for eTable 8.

**eTable 10. Cluster-level LOCF sensitivity analysis for HbA1c trajectories.**

| Group | Cluster | N | Δ (orig) | Δ (LOCF) | Δ diff | Max yr diff |
| --- | --- | --- | --- | --- | --- | --- |
| Monotherapy | 1 | 765 | -0.18 | -0.15 | 0.03 | 0.167 |
| Monotherapy | 2 | 707 | -0.04 | -0.07 | -0.03 | 0.236 |
| Monotherapy | 3 | 695 | -0.28 | -0.23 | 0.05 | 0.228 |
| Monotherapy | 4 | 363 | -0.1 | -0.15 | -0.05 | 0.45 |
| Monotherapy | 5 | 226 | -0.57 | -0.74 | -0.17 | 0.293 |
| Dual Therapy | 1 | 525 | -0.19 | -0.2 | -0.01 | 0.299 |
| Dual Therapy | 2 | 302 | -0.38 | -0.42 | -0.04 | 0.2 |
| Dual Therapy | 3 | 283 | -0.43 | -0.3 | 0.13 | 0.214 |
| Dual Therapy | 4 | 226 | -0.37 | -0.39 | -0.02 | 0.206 |
| Dual Therapy | 5 | 187 | -0.55 | -0.48 | 0.07 | 0.367 |
| Dual Therapy | 6 | 185 | -0.42 | -0.59 | -0.17 | 0.236 |
| Dual Therapy | 7 | 126 | -0.22 | -0.04 | 0.18 | 0.265 |
| Dual Therapy | 8 | 117 | -1.02 | -0.83 | 0.19 | 0.664 |
| Complex Therapy | 1 | 312 | -0.32 | -0.27 | 0.05 | 0.526 |
| Complex Therapy | 2 | 153 | -0.32 | -0.51 | -0.19 | 0.382 |
| Complex Therapy | 3 | 62 | -0.21 | -0.32 | -0.11 | 0.516 |
| Variant Therapy | 1 | 514 | -0.8 | -0.63 | 0.17 | 0.499 |
| Variant Therapy | 2 | 390 | -0.29 | -0.22 | 0.07 | 0.165 |
| Variant Therapy | 3 | 321 | -0.02 | -0.1 | -0.08 | 0.32 |
| GLP-1 Therapy | 1 | 628 | -0.47 | -0.37 | 0.1 | 0.219 |
| GLP-1 Therapy | 2 | 424 | -0.29 | -0.23 | 0.06 | 0.21 |
| GLP-1 Therapy | 3 | 333 | -0.38 | -0.36 | 0.02 | 0.139 |
| GLP-1 Therapy | 4 | 289 | -0.67 | -0.68 | -0.01 | 0.214 |
| GLP-1 Therapy | 5 | 271 | -0.2 | -0.45 | -0.25 | 0.334 |
| GLP-1 Therapy | 6 | 241 | -0.39 | -0.38 | 0.01 | 0.166 |
| GLP-1 Therapy | 7 | 187 | -0.45 | -0.46 | -0.01 | 0.263 |
| GLP-1 Therapy | 8 | 166 | -1.07 | -0.93 | 0.14 | 0.405 |
| GLP-1 Therapy | 9 | 143 | -0.99 | -1.01 | -0.02 | 0.169 |
| GLP-1 Therapy | 10 | 94 | -0.7 | -0.86 | -0.16 | 0.216 |
| GLP-1 Therapy | 11 | 87 | -1.19 | -1.1 | 0.09 | 0.514 |

**Note:** For each cluster, the table reports the within-cluster patient count (n), the modeled change in HbA1c from 2019 to 2024 under the original (complete-case) and LOCF analyses, the difference between the two estimates (Δ diff = Δ_LOCF − Δ_orig), and the maximum absolute difference in adjusted yearly means between the two analyses across 2019–2024 (Max yr diff). Adjusted estimates were derived from the manuscript's mixed-effects model specification refit on each dataset. Clusters are ordered by therapy group and within-group rank, consistent with Table 2.

**eTable 11. Cluster-level LOCF sensitivity analysis for BMI trajectories.**

| Group | Cluster | N | Δ (orig) | Δ (LOCF) | Δ diff | Max yr diff |
| --- | --- | --- | --- | --- | --- | --- |
| Monotherapy | 1 | 765 | -1.35 | -1.11 | 0.24 | 0.507 |
| Monotherapy | 2 | 707 | -1.31 | -1.29 | 0.02 | 0.2 |
| Monotherapy | 3 | 695 | -1.57 | -1.51 | 0.06 | 0.168 |
| Monotherapy | 4 | 363 | -1.08 | -1.19 | -0.11 | 0.247 |
| Monotherapy | 5 | 226 | -1.27 | -1.32 | -0.05 | 0.556 |
| Dual Therapy | 1 | 525 | -0.8 | -0.92 | -0.12 | 0.531 |
| Dual Therapy | 2 | 302 | -1.05 | -1 | 0.05 | 0.055 |
| Dual Therapy | 3 | 283 | -1.03 | -1.32 | -0.29 | 0.401 |
| Dual Therapy | 4 | 226 | -2.12 | -2.06 | 0.06 | 0.415 |
| Dual Therapy | 5 | 187 | -1.18 | -1.2 | -0.02 | 0.254 |
| Dual Therapy | 6 | 185 | -1.05 | -0.85 | 0.2 | 0.917 |
| Dual Therapy | 7 | 126 | 0.25 | 0.41 | 0.16 | 1.225 |
| Dual Therapy | 8 | 117 | -4.08 | -2.97 | 1.11 | 1.098 |
| Complex Therapy | 1 | 312 | -1.63 | -1.78 | -0.15 | 0.521 |
| Complex Therapy | 2 | 153 | -0.65 | -0.12 | 0.53 | 0.543 |
| Complex Therapy | 3 | 62 | 0.26 | -0.27 | -0.53 | 0.298 |
| Variant Therapy | 1 | 514 | -1.68 | -1.2 | 0.48 | 0.473 |
| Variant Therapy | 2 | 390 | -1.24 | -1.15 | 0.09 | 0.594 |
| Variant Therapy | 3 | 321 | -1.21 | -1.16 | 0.05 | 0.195 |
| GLP-1 Therapy | 1 | 628 | -2.47 | -2.34 | 0.13 | 0.641 |
| GLP-1 Therapy | 2 | 424 | -1.86 | -1.85 | 0.01 | 0.727 |
| GLP-1 Therapy | 3 | 333 | -1.76 | -1.78 | -0.02 | 0.748 |
| GLP-1 Therapy | 4 | 289 | -3.43 | -3.09 | 0.34 | 0.36 |
| GLP-1 Therapy | 5 | 271 | -2.02 | -1.61 | 0.41 | 0.523 |
| GLP-1 Therapy | 6 | 241 | -1.72 | -1.92 | -0.2 | 0.884 |
| GLP-1 Therapy | 7 | 187 | -2.49 | -2.57 | -0.08 | 0.805 |
| GLP-1 Therapy | 8 | 166 | -1.95 | -1.86 | 0.09 | 0.195 |
| GLP-1 Therapy | 9 | 143 | -1.79 | -1.75 | 0.04 | 0.334 |
| GLP-1 Therapy | 10 | 94 | -2.53 | -2.07 | 0.46 | 0.641 |
| GLP-1 Therapy | 11 | 87 | -2.27 | -2.41 | -0.14 | 1.024 |

**Note:** For each cluster, the table reports the within-cluster patient count (n), the modeled change in HbA1c from 2019 to 2024 under the original (complete-case) and LOCF analyses, the difference between the two estimates (Δ diff = Δ_LOCF − Δ_orig), and the maximum absolute difference in adjusted yearly means between the two analyses across 2019–2024 (Max yr diff). Adjusted estimates were derived from the manuscript's mixed-effects model specification refit on each dataset. Clusters are ordered by therapy group and within-group rank, consistent with Table 2.

**eFigure 1. Cohort Construction and Patient Selection**

Flow diagram showing inclusion and exclusion criteria used to derive the final study cohort. Adults (≥18 years) with type 2 diabetes (T2D) who were alive in 2019 and had at least one glucose-lowering medication prescription were identified. Patients were sequentially excluded based on encounter history, medication prescriptions, diagnosis timing, prior glucose-lowering therapy, evidence of uncontrolled hyperglycaemia, pregnancy-related diabetes (ICD-10 O24*), and residence outside the United States or unknown location. H1 and H2 denote the first (January–June) and second (July–December) halves of a calendar year, respectively.

**eFigure 2. Early Disengagement Cluster Visualizations.**

This eFigure presents two-panel cluster visualizations for patients classified as external to the primary analytic cohort. Early disengagement patients were identified as those assigned to clusters in which more than 70% of patients had medication data concentrated in the first 1–2 study intervals with minimal subsequent prescribing activity, consistent with care occurring substantially outside of TriNetX health systems. Each cluster visualization includes (1) individual prescribing trajectory heatmaps and (2) medication class prescribing over time. Because sustained longitudinal observation is required for trajectory analysis, these clusters were analyzed separately and are presented here to characterize distinct patterns of limited healthcare engagement. Abbreviations: CI, confidence interval; DPP-4i, dipeptidyl peptidase-4 inhibitor; F/U, follow-up; GIP/GLP-1 RA, dual glucose-dependent insulinotropic polypeptide and glucagon-like peptide-1 RA; GLP-1 RA, glucagon-like peptide-1 RA; INS, insulin; MET, metformin; NH, non-Hispanic; OR, odds ratio; SGLT2i, sodium-glucose cotransporter-2 inhibitor; SUL, sulfonylurea; TZD, thiazolidinedione.

**eFigure 3. Sensitivity Analysis: Therapy Group Concordance Under Alternative Filtering Approach.**

This eFigure presents the concordance between original therapy group assignments (post-hoc cluster-level filtering) and therapy group assignments derived from a sensitivity analysis using patient-level sparsity filtering prior to re-clustering. The heatmap displays row-normalized percentages: each cell shows the proportion of patients from a given original therapy group (rows) assigned to each sensitivity analysis group (columns). Diagonal values represent the concordance rate (the percentage of patients retaining the same therapy group classification). Re-clustering was performed using Ward's linkage with k = 40 on patients with ≤70% empty medication bins. Each new cluster's medication profile (proportion of patients on each drug class per time bin) was compared to the 30 original cluster profiles using cosine similarity and assigned to the therapy group of its closest match.

**eFigure 4. Sensitivity Analysis: Therapy Group Concordance Under 3-Month Binning.**

Concordance between original therapy group assignments (computed using 6-month bins) and therapy group assignments derived from re-clustering after rebuilding patient trajectories at 3-month resolution (24 quarterly bins, 2019–2024). The heatmap displays row-normalized percentages: each cell shows the proportion of patients from a given original therapy group (rows) assigned to each 3-month-binning therapy group (columns). Diagonal values represent concordance (the percentage of patients retaining the same therapy group classification). Re-clustering was performed using Ward's linkage with squared Euclidean distance on the full cohort (n = 18,652) at k = 40. Medication profiles for both new and original clusters were computed in the shared 6-month feature space (proportion of patients on each drug class per six-month bin); each new cluster was matched to the original cluster with the highest cosine similarity and inherited that cluster's therapy group label. Unlike the patient-level sparsity sensitivity analysis (**eFigure 3**), the Early Dropout group is included here because no patient-level sparsity filter was applied prior to re-clustering.

**eFigure 5. Sensitivity Analysis: Therapy Group Concordance Under Prescription-Time Alignment.**

Concordance between original therapy group assignments (computed from trajectories anchored to January 2019) and therapy group assignments derived from re-clustering after rebuilding patient trajectories anchored to each patient's individual first glucose-lowering medication prescription date. The heatmap displays row-normalized percentages: each cell shows the proportion of patients from a given original (calendar-time) therapy group (rows) assigned to each prescription-time therapy group (columns). Diagonal values represent concordance (the percentage of patients retaining the same therapy group classification); row and column ordering is determined by descending diagonal concordance. Re-clustering used Ward's linkage with squared Euclidean distance on the full cohort (n = 18,652) at k = 40. Trajectories were constructed using 11 six-month bins anchored at the patient's first prescription date (99-dimensional feature space) so that every patient contributed the same number of complete bins. Each new cluster was matched to the original cluster with the highest cosine similarity of medication profiles in the shared 99-dimensional feature space (11 six-month bins × 9 drug classes); original cluster centroids were truncated from 12 bins to 11 to occupy the same feature space as the prescription-time centroids. As in **eFigure 4**, the Early Dropout group is included because no patient-level sparsity filter was applied prior to re-clustering.

**eFigure 6. Vectorized Patient Prescribing Trajectory**

Schematic illustrating transformation of patient-level prescription histories into a binary feature matrix. Prescription events are aggregated into half-year intervals (H1: January–June; H2: July–December). Each row corresponds to a time interval and each column to a medication class, with matrix entries $M_{ij}=1$indicating that medication $j$was prescribed during interval $i$, and 0 otherwise. The resulting matrix is flattened into a fixed-length binary vector for clustering.

**eFigure 7. Comparison of Agglomerative Linkage Methods at k = 40.**

Each panel applies a different classical agglomerative linkage method to the same 18,652-patient input matrix using Euclidean distance: (A) single linkage, (B) complete linkage, (C) average linkage, and (D) Ward's linkage. Heatmap conventions match **eFigure 9**.

**eFigure 8. Internal Validity Diagnostics for Selecting the Number of Clusters (k).**

Two complementary criteria for choosing k are presented across k = 2 to 50, computed using Ward's linkage with squared Euclidean distance on the full clustering input matrix (n = 18,652 patients × 108-dimensional prescribing trajectory vectors). (A) Within-cluster sum of squares (WCSS), used in the elbow method. (B) Gap statistic, computed against B = 10 uniform-random reference. The selected value k = 40 is marked in both panels.

**eFigure 9. Patient-Level Prescribing Trajectory Heatmaps Across Increasing Values of k.**

Each panel shows the same 18,652 patients clustered with Ward's linkage and squared Euclidean distance at k = 20, 30, 40, and 50. Each row of each heatmap is one patient; columns are six-month bins from 2019-H1 through 2024-H2; colors encode medication class as shown in the legend. Black horizontal lines separate clusters. **Abbreviations:** DPP-4i, dipeptidyl peptidase-4 inhibitor; GIP/GLP-1 RA, dual glucose-dependent insulinotropic polypeptide and glucagon-like peptide-1 RA; GLP-1 RA, glucagon-like peptide-1 RA; INS, insulin; MET, metformin; SGLT2i, sodium-glucose cotransporter-2 inhibitor; SUL, sulfonylurea; TZD, thiazolidinedione.

**eFigure 10. Comprehensive Dual Therapy Cluster Visualizations.**

This eFigure presents three-panel visualizations for all clusters classified within the Dual Therapy group. Each panel includes (1) individual patient trajectory heatmaps, (2) medication prescribing distributions over time, and (3) adjusted BMI and HbA1c trajectories where available. A subset of representative clusters from this group is displayed in the main manuscript figures, while this eFigure provides visualizations for all clusters in this group. **Abbreviations:** **CI**, confidence interval; **DPP-4i**, dipeptidyl peptidase-4 inhibitor; **F/U**, follow-up; **GIP/GLP-1 RA**, dual glucose-dependent insulinotropic polypeptide and glucagon-like peptide-1 RA; **GLP-1 RA**, glucagon-like peptide-1 RA; **INS**, insulin; **MET**, metformin; **NH**, non-Hispanic; **OR**, odds ratio; **SGLT2i**, sodium-glucose cotransporter-2 inhibitor; **SUL**, sulfonylurea; **TZD**, thiazolidinedione.

**eFigure 11. Complex and Variant Therapy Cluster Visualizations.**

This figure presents three-panel visualizations for all clusters classified within the Complex and Variant therapy groups**.** Each panel includes (1) individual patient trajectory heatmaps, (2) medication usage distributions over time, and (3) adjusted BMI and HbA1c trajectories where available. **Abbreviations:** **CI**, confidence interval; **DPP-4i**, dipeptidyl peptidase-4 inhibitor; **F/U**, follow-up; **GIP/GLP-1 RA**, dual glucose-dependent insulinotropic polypeptide and glucagon-like peptide-1 RA; **GLP-1 RA**, glucagon-like peptide-1 RA; **INS**, insulin; **MET**, metformin; **NH**, non-Hispanic; **OR**, odds ratio; **SGLT2i**, sodium-glucose cotransporter-2 inhibitor; **SUL**, sulfonylurea; **TZD**, thiazolidinedione.

**eFigure 12. Comprehensive GLP-1 Therapy Cluster Visualizations (clusters 1-6).**
This eFigure presents three-panel visualizations for clusters 1–6 within the GLP-1 therapy group. Each panel includes (1) individual patient trajectory heatmaps, (2) medication usage distributions over time, and (3) adjusted BMI and HbA1c trajectories, where available. Representative clusters from this group are shown in the main manuscript figures, while this eFigure provides comprehensive visualizations for the first six clusters to ensure transparency of cluster-level patterns. **Abbreviations:** **CI**, confidence interval; **DPP-4i**, dipeptidyl peptidase-4 inhibitor; **F/U**, follow-up; **GIP/GLP-1 RA**, dual glucose-dependent insulinotropic polypeptide and glucagon-like peptide-1 RA; **GLP-1 RA**, glucagon-like peptide-1 RA; **INS**, insulin; **MET**, metformin; **NH**, non-Hispanic; **OR**, odds ratio; **SGLT2i**, sodium-glucose cotransporter-2 inhibitor; **SUL**, sulfonylurea; **TZD**, thiazolidinedione.

**eFigure 13. Comprehensive GLP-1 Therapy Cluster Visualizations (clusters 7-11).**
This eFigure presents three-panel visualizations for clusters 7–11 within the GLP-1 therapy group. Each panel includes (1) individual patient trajectory heatmaps, (2) medication usage distributions over time, and (3) adjusted BMI and HbA1c trajectories, where available. Representative clusters from this group are shown in the main manuscript figures, while this eFigure provides comprehensive visualizations for the last five clusters to ensure transparency of cluster-level patterns. Abbreviations: CI, confidence interval; DPP-4i, dipeptidyl peptidase-4 inhibitor; F/U, follow-up; GIP/GLP-1 RA, dual glucose-dependent insulinotropic polypeptide and glucagon-like peptide-1 RA; GLP-1 RA, glucagon-like peptide-1 RA; INS, insulin; MET, metformin; NH, non-Hispanic; OR, odds ratio; SGLT2i, sodium-glucose cotransporter-2 inhibitor; SUL, sulfonylurea; TZD, thiazolidinedione.

**eFigure 14. Distribution of Higher-Level Prescribing Trajectory Categories by Race/Ethnicity and by HF/CKD Status.** Each bar shows the percentage of patients within a given stratum (race/ethnicity in Panel A; composite HF/CKD status in Panel B) assigned to each of the five higher-level prescribing trajectory categories defined in eTable 3 (Monotherapy, Dual Therapy, Complex Therapy, Variant Therapy, GLP-1 Therapy). Bars sum to 100% within each stratum; stratum-level denominators are shown beneath each x-axis label. The composite HF/CKD indicator follows the same definition used elsewhere in the analysis (documented heart failure or chronic kidney disease between January 1 and June 1, 2019). The figure reflects the primary analytic cohort; early-disengagement clusters are analyzed separately (eFigure 2).

**eFigure 15.** **Sensitivity Analysis: BMI and HbA1c Trajectories Under LOCF Imputation.**

Overlay of original (complete-case) and LOCF-imputed adjusted BMI and HbA1c trajectories for representative clusters spanning the five therapy groups (Monotherapy – Cluster 1, Dual Therapy – Cluster 1, Complex Therapy – Cluster 1, Variant Therapy – Cluster 1, GLP-1 Therapy – Cluster 1). Solid blue lines show the original analysis; dashed orange lines show the LOCF sensitivity analysis. Visual concordance between the two trajectories supports robustness of the manuscript's primary findings to relaxed measurement-completeness requirements.

**eFigure 16. Cluster-Level Patient Characteristics — Standardized View.**

This eFigure presents the manuscript Table 1 characteristics for each of the 30 primary clusters as a heatmap. Rows are clusters, ordered by therapy group to match manuscript Tables 2 and 3, with horizontal lines demarcating group boundaries. Columns are mean age, % female, the racial/ethnic distribution, mean baseline HbA1c and BMI, and the prevalence of documented HF and CKD. Each cell is annotated with the raw value, and shaded by the per-column z-score across the 30 clusters: red indicates values above the cluster-level mean, blue indicates values below, and saturation reflects the absolute z-score on a shared scale centered at zero. Standardization is applied per column so that variables on different native scales remain visually comparable. Abbreviations: BMI, body mass index; CKD, chronic kidney disease; DPP-4i, dipeptidyl peptidase-4 inhibitor; F/U, follow-up; GIP/GLP-1 RA, dual glucose-dependent insulinotropic polypeptide and glucagon-like peptide-1 RA; GLP-1 RA, glucagon-like peptide-1 RA; HbA1c, glycated hemoglobin; HF, heart failure; INS, insulin; MET, metformin; NH, non-Hispanic; SGLT2i, sodium-glucose cotransporter-2 inhibitor; SUL, sulfonylurea; TZD, thiazolidinedione.
